# Age-related meningeal extracellular matrix remodeling compromises CNS lymphatic function

**DOI:** 10.1186/s12974-025-03436-0

**Published:** 2025-04-17

**Authors:** Kate Hitpass Romero, Taylor J. Stevenson, Leon C. D. Smyth, Ben Watkin, Samuel J.C. McCullough, Luca Vinnell, Amy M. Smith, Patrick Schweder, Jason A. Correia, Jonathan Kipnis, Mike Dragunow, Justin Rustenhoven

**Affiliations:** 1 Department of Pharmacology and Clinical Pharmacology, The University of Auckland, Auckland, New Zealand; 2 Centre for Brain Research, The University of Auckland, Auckland, New Zealand; 3 Brain Immunology and Glia Center, School of Medicine, Washington University in St. Louis, St. Louis, MO USA; 4 Department of Pathology and Immunology, School of Medicine, Washington University in St. Louis, St. Louis, MO USA; 5 Immunology Graduate Program, School of Medicine, Washington University in St. Louis, St. Louis, MO USA; 6 Auckland City Hospital, Auckland, 1023 New Zealand

**Keywords:** Fibrosis, Lymphatics, Aging, Traumatic brain injury, Extracellular matrix, Meninges, Meningeal immunity

## Abstract

Efficient clearance of central nervous system (CNS) waste proteins and appropriate immune surveillance is essential for brain health. These processes are facilitated by lymphatic networks present in the meninges that drain cerebrospinal fluid (CSF). Age-related impairments to meningeal lymphatic drainage contribute to CNS waste accumulation and immune dysfunction, yet the underlying mechanisms remain poorly understood. Here, we identify extracellular matrix (ECM) remodeling in the aged dura as a key driver of CSF clearance deficits, demonstrating that peri-lymphatic collagen accumulation disrupts lymphatic function. Exploring immune-derived factors contributing to this ECM remodeling, we identify transforming growth factor beta 1 (TGFβ1) as a major regulator using primary human dural fibroblasts. Using a novel mouse model with constitutively active TGFβ receptor 1 (TGFβR1) signaling in dural fibroblasts, we show that excessive peri-lymphatic collagen deposition impairs meningeal lymphatic drainage and alters meningeal immunity. Mechanistically, we reveal that ECM-associated matrix stiffness disrupts lymphatic junction integrity and impairs lymphangiogenesis in human lymphatic endothelial cells. These findings establish dural immune cell and fibroblast-mediated ECM remodeling as a critical regulator of CSF clearance and highlight it as a potential therapeutic target for restoring brain waste clearance in aging.

**Supplementary Information:**

The online version contains supplementary material available at 10.1186/s12974-025-03436-0.

## Background

Efficient clearance of metabolic waste and neurotoxic proteins from the central nervous system (CNS) is crucial for maintaining brain health and function [1, 2]. In peripheral tissues, waste removal is achieved by tissue-resident lymphatic systems that maintain homeostatic functions by draining interstitial fluid and facilitating immune surveillance [3]. The brain is distinct in that it lacks a parenchymal lymphatic network. Instead, fluid and waste removal from the CNS is facilitated by two connected systems, the glymphatic system and lymphatic networks in the brain border tissues—the meninges [2]. Together, these CNS clearance routes shuttle waste-laden interstitial fluid out of the brain, into the cerebrospinal fluid (CSF), to the dural layer of the meninges via discontinuities in the arachnoid layer, and then to deep cervical lymph nodes (dCLNs) via a dural lymphatic network [4–8]. Recently, an additional site for CSF drainage via a lymphatic plexus in the nasopharynx was described that also permits drainage of CSF to dCLNs [9]. Collectively, these networks enable effective CNS waste clearance.

While clearance of CSF, and by extension, the removal of brain interstitial fluid, is crucial for homeostatic CNS function, it is even more important during varied insults which generate excessive pathological waste. These conditions include age-related neurodegenerative diseases, such as Parkinson’s and Alzheimer’s disease, which are characterized by the accumulation of misfolded proteins within the CNS and CSF that drive neuroinflammation and neuronal loss [10]. Similarly, traumatic brain injuries (TBIs) result in the generation of hyperphosphorylated tau species, cellular debris, and toxic blood-derived products which can also disrupt neuronal function [11]. Critically, aging, age-related neurodegenerative diseases, and TBIs are all associated with compromised CNS-draining lymphatic function, further exacerbating neurological dysfunction [8, 12–14]. While the exact cause(s) of these CSF clearance deficits are unclear, restoring CSF drainage improved neurological outcomes in both age-related neurodegenerative diseases and TBIs [12, 13]. However, such approaches use proof-of-concept interventions that do not target the underlying pathology and are not amendable to rapid clinical translation or acute deployment. Understanding the mechanisms driving CSF clearance failure is needed to develop effective therapeutics.

Recently, we and others described a common pathology across TBIs and aging that could contribute to meningeal lymphatic decline, namely dural extracellular matrix (ECM) remodeling [15, 16]. This remodeling is marked by excessive deposition of ECM components—particularly type 1 collagen—around dural lymphatic networks in aging and TBIs. In peripheral tissues, such fibrosis or ECM remodeling impairs lymphatic function by disrupting parenchymal tissue architecture and increasing ECM rigidity [17–20]. The ECM is a complex network of structural and bioactive molecules, including collagens, glycoproteins, and proteoglycans. Excessive ECM remodeling can lead to fibrosis, characterized by the accumulation of connective tissue, stromal hardening, and scar formation. Similarly, desmoplasia, refers to the proliferation of fibrous tissue in response to tissue injury such as cancer or infection. These processes involve increased ECM protein deposition, cross-linking, and structural reorganization, which contribute to tissue stiffening and functional impairment [21, 22]. Different collagen subtypes have distinct roles in tissue mechanics and fibrosis. Type I collagen, including Col1a1—primarily produced by fibroblasts—is the main structural protein in connective tissues, providing tensile strength and contributing to tissue stiffness [23, 24]. In contrast, type III collagen is more flexible, affecting elasticity, while type IV collagen is a major component of basement membranes supporting cellular adhesion and filtration. Col1a1 expression strongly correlates with tissue stiffness, enhancing matrix rigidity and impairing cellular functions and tissue homeostasis in fibrotic and aged tissues [24]. Other ECM components, such as fibronectin and laminin, also undergo remodeling during fibrosis, influencing cellular adhesion, signaling, and matrix organization.

Here, we demonstrate that age-related ECM remodeling hinders the functionality of meningeal lymphatic vessels and exacerbates waste clearance deficits. We find that the aged dura mater displays collagen reorganization to peri-lymphatic regions, analogous to that seen in the aged skin [25]. Investigating age-related immune changes driving this phenotype, we identify TGFβ1 as a key factor in driving collagen deposition from human dural fibroblasts. Using a novel mouse model of dural fibrosis through delivery of a mutated constitutively active TGFβ receptor to dural fibroblasts—via CSF-delivered adeno-associated viruses (AAVs)—we demonstrate that enhanced collagen deposition around the meningeal lymphatics impairs lymphatic function and alters meningeal immune populations. Finally, using induced pluripotent stem cells (iPSC)-derived lymphatic endothelial cell (LEC) models, we demonstrate that ECM-related elevations in tissue stiffness hinders key lymphatic phenotypes critical to CSF drainage, including disrupting junctional proteins and impairing lymphangiogenesis. Collectively, these data highlight how ECM changes in the dura mater can compromise CSF clearance and suggest the exploration of anti-fibrotic therapies or immunomodulatory strategies as promising interventions to normalize CSF clearance in neurological disorders.

## Results

### ECM alterations in the aged dura mater

**Fig. 1:**
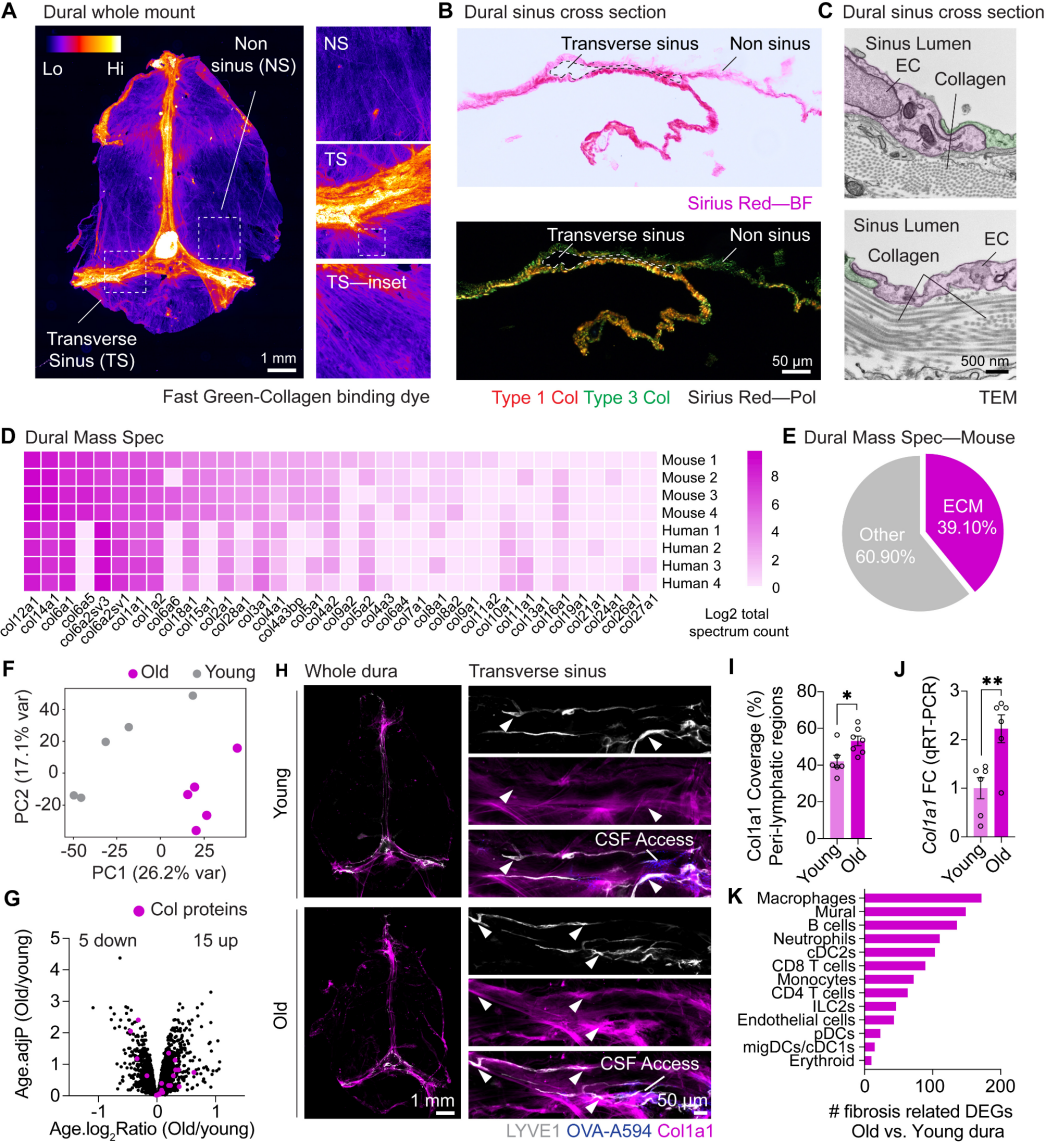
**Collagen alterations in the aged dura. (A) **Dural whole mount stained with Fast Green dye indicating collagen-dense regions. Insets represent enlarged regions of non-sinus and transverse sinus regions. **(B)** Sirius Red staining of a dural sinus cross-section, with collagen type I/III visualized by polarized light. **(C)** Transmission electron microscopy (TEM) demonstrating composition of mouse dural sinus including endothelial cells (ECs) lining the lumen and collagen bundles. **(D)** Unlabeled Liquid chromatography-mass spectrometry (LC-MS) analysis for collagen subtype expression in human and mice dural homogenates, *n* = 4 dural samples per species. **(E)** Pie chart demonstrating the abundance of extracellular matrix (ECM) proteins that make up the mouse dura from mass spectrometry analysis. **(F) **Principal component analysis plot for dural protein expression determined by Tandem Mass Tag (TMT)-labeled LC-MS analysis from young (2-3 months) and old (20-24 months) mice showing relative changes in collagen proteins, *n* = 5 dural samples per age. **(G)** Volcano plot of average log_2_ fold change and -log_2_ adjusted p value for alterations in old and young dural proteins. Collagen proteins are coloured in magenta. Data points represent the average of *n* = 5 mice per age. **(H)** Immunohistochemistry of Col1a1 coverage in dural whole mounts and the peri-lymphatic regions of the transverse sinus regions of young (2-3 months) and old (20-24 months) mice. An intra-cisterna magna injection of OVA-A594 (2.5 μL OVA-A594 1 µg/mL) was performed one hour prior to euthanasia, indicating sites of CSF access in the transverse sinus region. Arrowheads highlight peri-lymphatic collagen. (**I**) Quantification of Col1a1 coverage in the peri-lymphatic regions of the transverse sinus. *, *P *< 0.05 (Student’s t test), *n* = 6 young mice, *n* = 7 old mice.** (J) **qPCR analysis of Col1a1 expression in the dura of young and old mice. **, *P* < 0.01 (Student’s t test), *n* = 6 per age. **(K)** Analysis of significant differentially expressed genes (DEGs) relating to fibrosis pathways, using the curated gene list from Fibroatlas, in old mouse dura compared to young mouse dura by cell type. Data are from scRNA-seq analysis of young and old whole dura from [16]

To identify whether ECM alterations may contribute to CSF clearance deficits, we first sought to better characterize the homeostatic ECM environment of the dura mater. Because collagens, and particularly type 1 collagen, are major regulators of tissue stiffness and fibrosis [23, 24], we chose to focus on collagen components. Under steady state conditions the dura mater is highly collagenous, providing physical support to the brain parenchyma. Using Fast Green (a collagen-binding dye) on mouse dural whole mounts, collagen fibers were observed in all regions of the dura mater but were particularly enriched around the dural sinuses where the meningeal lymphatics are located [6] (Fig. 1A). Next, we used Sirius Red staining—which enables the visualization of different collagen types under polarized light—to reveal an enrichment of type 1 collagen around the dural sinuses (Fig. 1B). Using transmission electron microscopy (TEM) we observed dense networks of aligned and disorganized collagen fibers surrounding the dural sinus lumen (Fig. 1C). Finally, using unlabeled liquid-chromatography-mass spectrometry (LC-MS), we observed 36 distinct collagen isoforms in the young mouse dura, 30 of which were conserved in the human dura (Fig. 1D), and ECM-related proteins were responsible for 39.1% of all peptides observed in mouse dura homogenates (Fig. 1E).

Type 1 collagen is a major contributor to the overall stiffness of a tissue, and a predominant mediator of tissue fibrosis [24]. While an increase in dural sinus-associated collagen type I was previously reported in the dura of aged mice or those suffering a TBI [15, 16], the full complexity of ECM alterations during aging remains unclear. To quantify the relative abundance of all dural collagen components, we conducted tandem mass tag (TMT)-labeled LC-MS of dural homogenates from young and aged mice. Dimensionality reduction of these data via Principal Component Analysis (PCA), demonstrated segregation of the overall dural proteome by age (Fig. 1F). Collagen peptides demonstrated a heterogeneous response in collagen protein abundance between old (20–24 months) and young (2–3 months) dural samples (Fig. 1G), with 15 being upregulated—including Col1a1—and 5 displaying a downregulation.

While LC-MS analysis is beneficial for determining holistic changes in ECM quantities, it is less useful for understanding spatial distributions. For example, during aging, the skin dermis shows a loss of collagen abundance, but an elevated expression around lymphatic networks, partially because of elevated immune cell infiltration or retention [25, 26]. As we previously demonstrated age-related accumulation of immune cells around the dural sinuses [16], we hypothesized these may drive collagen accumulation in these regions which would have the greatest impact on CSF drainage. As such, we analyzed collagen distribution around lymphatic networks in the transverse sinus—sites where CSF access to the dura is observed—in young and aged mice injected with 40 kDa fluorescent ovalbumin (OVA-A594) into the CSF. Analogous to that seen in the aged skin, a redistribution of Col1a1 was observed, displaying increased abundance in peri-lymphatic regions (Fig. 1H and I). Elevated expression of *Col1a1* transcripts were also observed in the aged dura mater (Fig. 1J). Finally, to determine age-related transcriptional changes in fibrosis pathways within the dura mater that may be responsible for these changes, we performed differential expression analysis of fibrosis-related genes—using a curated list from FibroAtlas [27]—across various cell types in aged versus young mouse dura single cell RNA-sequencing (sc-RNA-seq) datasets [16]. Macrophages and mural cells exhibited the highest number of fibrosis-related differentially expressed genes, indicating significant age-related transcriptional changes in these cell types (Fig. 1K). Overall, these findings indicate functional and structural re-organization of Col1a1 during aging that may contribute to the age-related decline in CSF drainage.

### Identifying drivers of dural ECM remodeling

**Fig. 2:**
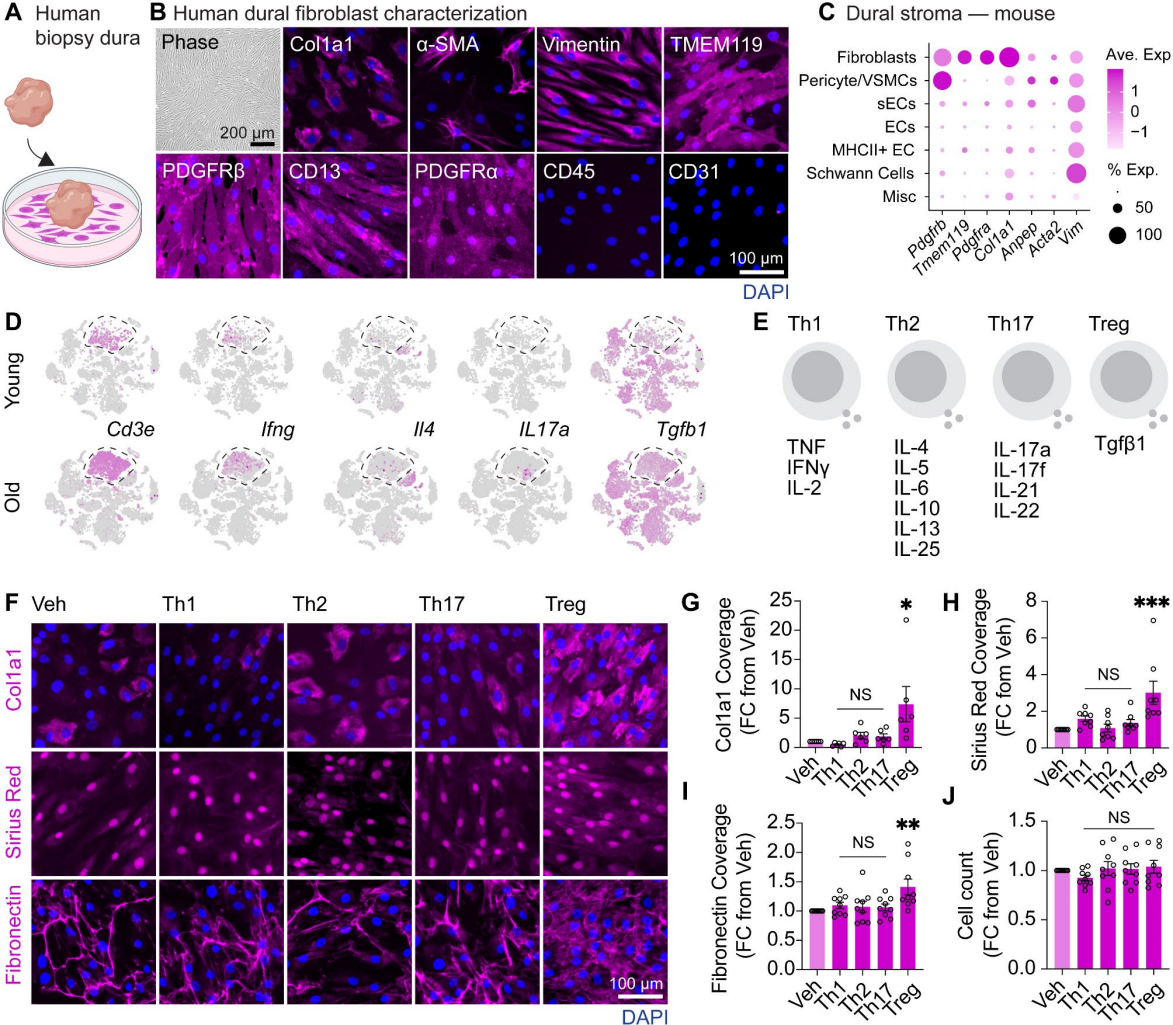
**TGFβ1 promotes a fibrotic response in vitro in human dural fibroblasts. (A) **Schematic for the growth of human dural fibroblasts obtained from tissue biopsies.** (B) **Phenotypic characterization of dural fibroblast cultures demonstrating expression of Col1a1, α-SMA, vimentin, TMEM119, PDGFRβ, CD13 and PDGFRα and absence of CD45 and CD31. Images are representative of *n* = 3 independent cases. **(C) **Dot plot showing scaled expression and percentage of cells expressing these genes in mouse dural stroma populations from reanalysis of scRNA-seq data in [16]. **(D) **tSNE visualization of scRNA-seq data from young and old whole dura samples by [16] showing the *Cd3e* T cell cluster, and presence of Th1, Th2, Th17 and Treg cells determined by expression of *Ifng*, *Il4*, *Il17a*, and *Tgfb1* respectively, *n* = 5 individual young and old dura per experiment, *n* = 2 independent experiments, 10 dura samples per age total.**(E) **Schematic of the cytokine cocktails corresponding to different secretion patterns of T cell polarized states, namely Th1, Th2, Th17, and Treg. **(F-J) **Immunocytochemistry and quantification of Col1a1, Sirius Red, and fibronectin expression in human dural fibroblast cultures after treatment with Veh, Th1, Th2, Th17 or Treg cytokine cocktail at 10 ng/mL for 48 hours. *, *P* < 0.05; **, *P* < 0.01; ***, *P* < 0.001 One-way ANOVA with Kruskal-Wallis post-hoc test, *n* = 6 independent human dural cultures for Col1a1, *n* = 8 independent human dural cultures for Sirius Red, *n* = 9 independent human dural cultures for fibronectin

Fibroblasts are the major contributors to collagen deposition in most tissues and our reanalysis of whole-dura scRNA-seq data confirmed that dural fibroblasts were primarily responsible for collagen expression in the dura [16] (Fig. S1 A-D). To determine factors that may contribute to age-related dural collagen deposition, we first developed and characterized explant-based methods to expand dural fibroblasts from human patient biopsy samples (Fig. 2A). Characterizing these human dural fibroblasts against known markers for dural fibroblasts from mice demonstrated expression of Col1a1, α-SMA, vimentin, TMEM119, PDGFRβ, CD13 and PDGFRα and absence of hematopoietic and endothelial cell markers CD45 and CD31, respectively (Fig. 2B and C).

Fibrosis is a complex phenomenon consisting of multiple diverging pathways and cellular contributors [23]. One important contribution comes from the innate and adaptive arms of the immune system, particularly via cytokine secretion. Peri-lymphatic T cell accumulation has been suggested to contribute to the altered collagen abundance in the skin [25], and indeed we previously described T cell accumulation in the aged dura, particularly around the dural sinuses. These cells are a source of multiple cytokines which can participate in fibrotic responses [16]. In the meninges, all classical Th1, Th2, Th17, and Treg subsets are present [16] (Fig. 2D) and as such we queried whether T cell-derived cytokines were an important contributor to age-related dural fibrosis. To achieve this, human dural fibroblasts were treated with cytokine cocktails corresponding to the different secretion patterns of T cell polarized states (Fig. 2E) and ECM proteins Col1a1, fibronectin, and total collagen deposition via Sirius Red, was examined (Fig. 2F). Interestingly, despite evidence for Th2 and Th17-derived cytokines driving fibrotic responses [23], only TGFβ1 induced significant changes in ECM production (Fig. 2G-J). Expansion of Tregs, which are major producers of TGFβ1 is observed in the aged meninges [28], though it should be noted numerous other immune and stromal cells can also contribute to this production. In agreement with these findings, reanalyzing age-related transcriptional responses in mouse dural fibroblasts [16] revealed a significant enrichment of gene ontology pathways relating to biological response to TGFβ1 (Fig. S1, E and F). Similar findings were observed previously in TBI models, where ligand-receptor interaction networks predicted TGFβ1 as the major driver of TBI-induced fibrosis [15, 29] and CSF levels of TGFβ1 are positively correlated with biological age in human proteomic datasets [30]. These results suggest that specific cytokine-driven polarization states—particularly TGFβ1—can modulate ECM production from dural fibroblasts, indicating potential mechanistic pathways for dural fibrosis and immune interactions.

### Modeling dural ECM deposition in mice

**Fig. 3:**
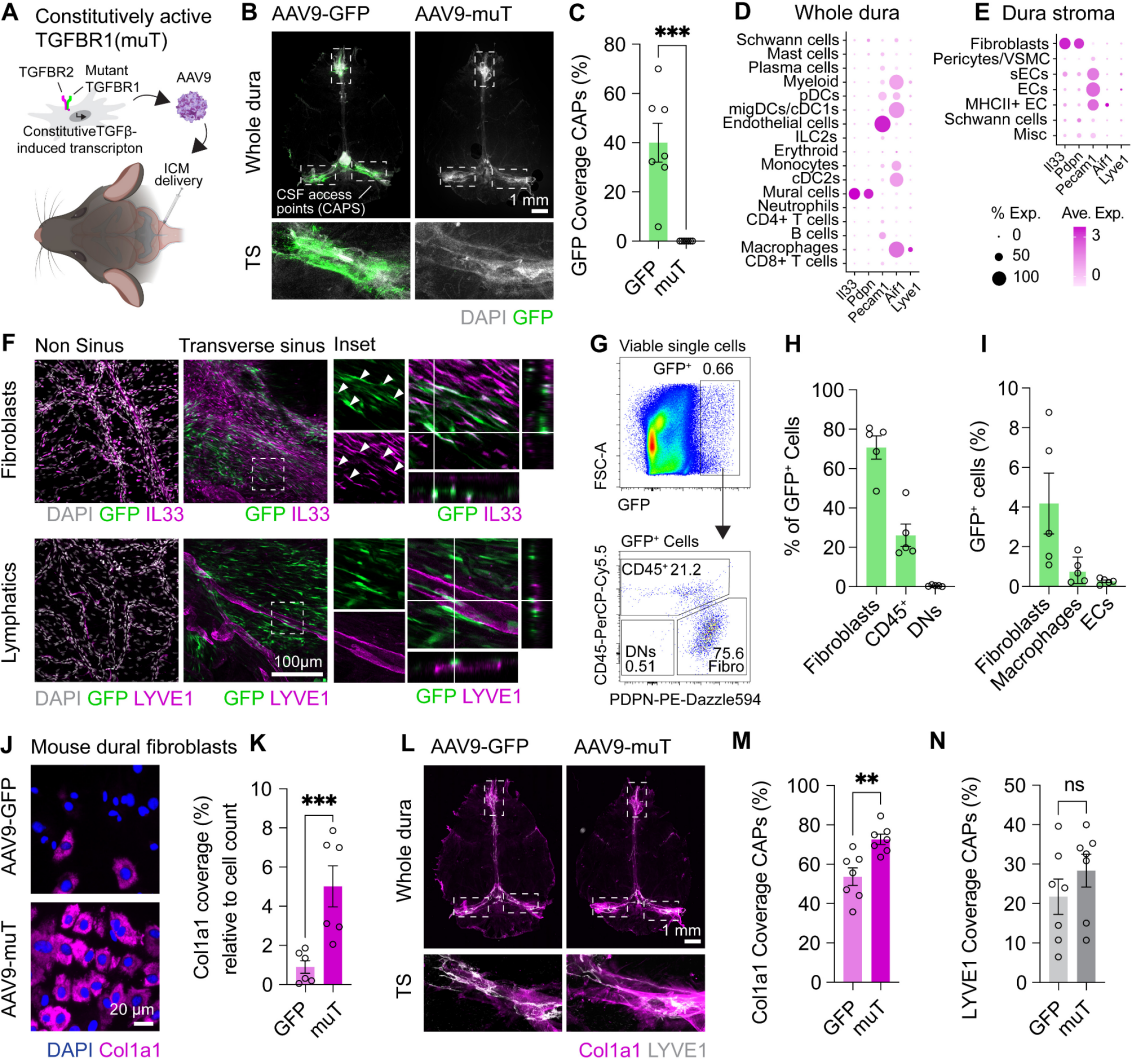
**Modeling dural ECM deposition using a constitutively active TGFβR1. (A) **Schematic depicting our fibrosis model via the delivery of an AAV9 carrying a constitutively active TGFβR1 (muT) to the CSF-filled cisterna magna. **(B)** Whole mount mouse dura depicting GFP coverage surrounding CSF access points (CAPs) one month after CSF delivery of AA9-CMV-GFP or muT (3 μL of 1x10^13^ GC/mL). Insets represent dotted box regions. **(C) **Quantification of GFP coverage in CAPs of GFP and muT mice. ***, *P* < 0.001 (Student’s t test), *n* = 7 mice per AAV. **(D) **Dot plot demonstrating scaled gene expression and percentage of cells expressing these genes for cell-specific markers in populations from the whole dura or** (E)** in dura stromal populations from scRNA-seq analysis by [16]. **(F)** Confocal images of non-sinus and sinus region showing GFP expression in IL33^+^ fibroblasts around the transverse sinus but not non sinus regions one month after CSF delivery of AA9-CMV-GFP or muT (3 μL of 1x10^13^ GC/mL).**(G) **Flow cytometry gating strategy for the identification of GFP expressing cells in the whole dura, one month after delivery of AA9-CMV-GFP or muT (3 μL of 1x10^13^ GC/mL). **(H) **Quantification of the proportions of fibroblasts, CD45^+^ cells or double negative (DNs) cells within the GFP^+^ gate. (**I**) Quantification for GFP expression within CD45^-^CD31^-^PDPN^+^ fibroblasts, CD45^+^CD11b^+^Ly6g^-^Ly6c^-^F4/80^+^ macrophages, or CD45^-^CD31^+^ endothelial cells. **(J, K)** Immunocytochemistry and quantification for Col1a1 expression one week following AAV9 transduction with 1x10^10^ GC/mL AAV9-GFP or AAV9-muT. **, *P* < 0.01 (Student’s t test), *n* = 6 independent mouse dura cultures per group. **(L)** Whole mount mouse dura depicting Col1a1 coverage surrounding CAPs one month after CSF delivery of AA9-CMV-GFP or muT (3 μL of 1x10^13^ GC/mL). Insets represent regions outlined by dotted box. **(M, N) **Quantification of Col1a1 and Lyve1 coverage in CAPs of GFP and muT mice. ***, *P* < 0.001 (Student’s t test), *n* = 7 mice per AAV

To determine whether dural ECM deposition around meningeal lymphatics contributes to age-related CSF clearance deficits, we sought to model this process in mice. Given prior evidence implicating TGFβ1 in aging and TBI-related dural fibrosis, we targeted this pathway. However, to distinguish the direct effects of fibrosis from indirect effects of TGFβ1 signaling on lymphatics, we opted to not simply overexpress TGFβ1, but instead deliver a mutated constitutively active TGFβ1 receptor (muT) to dural fibroblasts. This receptor carries three missense mutations: T204D that constitutively activates the TGFβR1 kinase and L193A/P194A which restrict binding of FKBP12, an endogenous TGFβR1 inhibitor, resulting in constitutively activated TGFβR1 signaling in cells [31, 32]. This enabled us to drive downstream signaling independently of the endogenous ligand (Fig. S2, A-C). To validate this model, we first cultured mouse dural fibroblasts and constructed AAVs with serotype 9 carrying either green florescent protein (GFP) or muT under the cytomegalovirus (CMV) promoter. In vitro analysis confirmed efficient transduction of mouse dural fibroblasts with AAV9-GFP (Fig. S2 D-H). To assess whether this approach enabled transduction of dural fibroblasts in vivo, we delivered the AAV9-GFP or muT into the CSF via an intra-cisterna magna injection (Fig. 3A). One month following AAV9 delivery, GFP expression was restricted to the CSF access points (CAPs)—specific sites that allow CSF to access the dura from the subarachnoid space due to discontinuities in the otherwise impermeable arachnoid barrier [5, 16] (Fig. 3B-C). To determine the specificity of cell transduction, we utilized immunostaining to cell-specific markers, including IL33 for dural fibroblasts, IBA1 for dural macrophages, CD31 (*Pecam1*) for dural endothelial cells, or Lyve1 for dural lymphatics (Fig. 3D-F and Fig. S2 I). GFP transduction was observed in dural fibroblasts, but not blood or lymphatic vasculature, specifically confined to dural sinus regions. Flow cytometry further confirmed this pattern, showing GFP expression predominantly in dural fibroblasts, with a smaller number of macrophages also showing transduction (Fig. 3G-I and Fig. S2J). Collectively, these data demonstrate that CSF-injection of an AAV9-CMV serotype/promotor combination efficiently targets dural fibroblasts.

Next, we sought to determine whether dural fibroblast transduction with the AAV9-muT model drives peri-lymphatic dural ECM deposition. We first examined Col1a1 expression in mouse dural fibroblasts in vitro one week following AAV9 delivery. Col1a1 was upregulated in fibroblasts transduced with AAV9-muT compared to GFP control (Fig. 3J-K). Similar findings were observed in vivo, where whole-mount imaging one month post injection of AAV9s into the CSF revealed that muT transduced mice displayed a marked increase in Col1a1 deposition around lymphatics at CAPs, comparable to what was observed during aging (Fig. 3L-N). To determine the specificity of this phenotype we examined GFP expression and Col1a1 induction in parenchymal brain tissue, leptomeninges, choroid plexus, and dCLNs, all of which showed no evidence for AAV transduction or Col1a1 alterations (Fig. S3 A-H). These results demonstrate that overexpression of constitutively active TGFβR1 in dural fibroblasts via CSF delivery induces Col1a1 deposition around meningeal lymphatics.

### Dural ECM remodeling impairs CSF clearance pathways

**Fig. 4:**
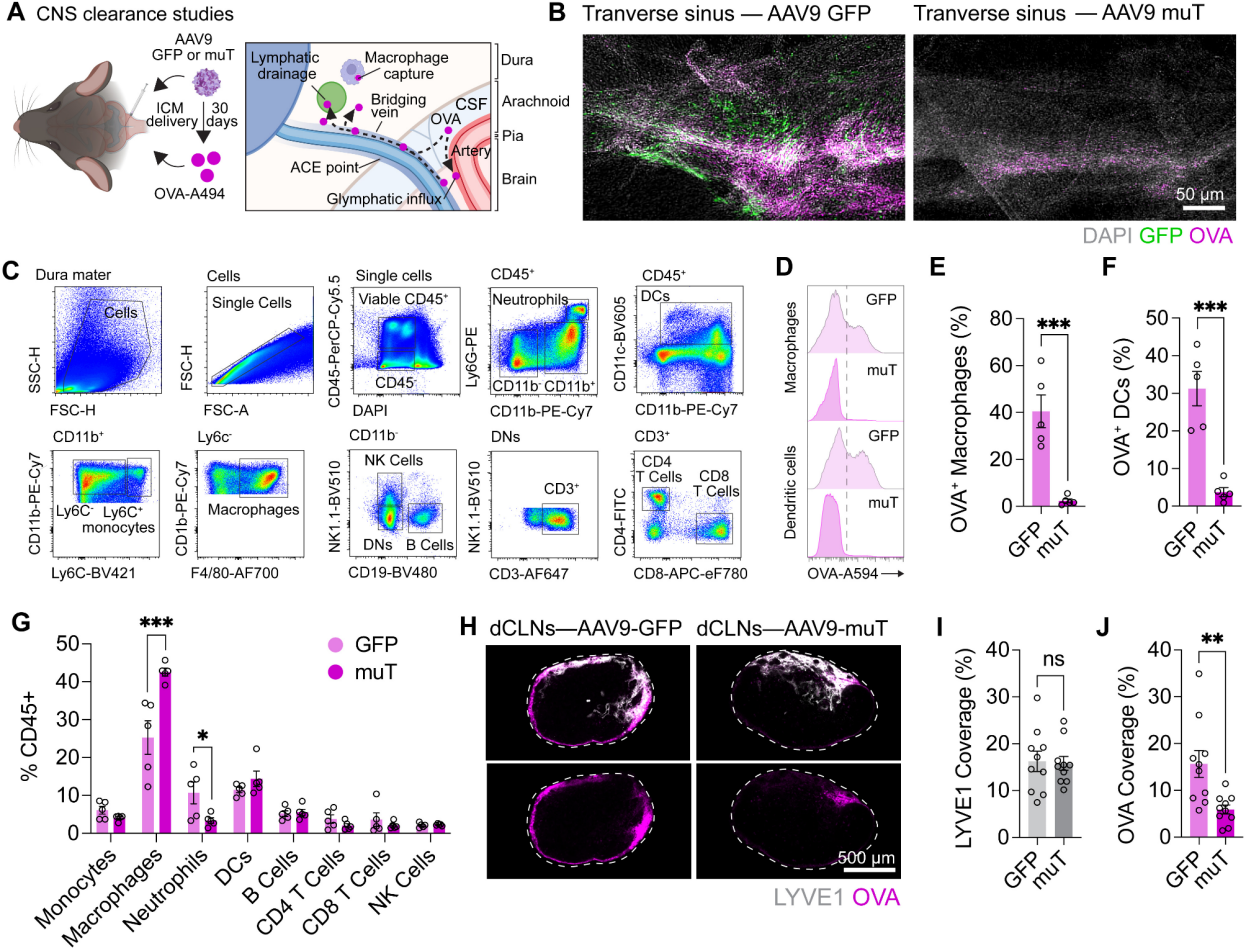
**Dural ECM deposition impairs CSF clearance. (A) **Schematic representing the proposed model to promote dural fibrosis via CSF-delivery of AAVs and assess CSF clearance one month later (lymphatic drainage, glymphatic influx, and CSF efflux). **(B) **Confocal imaging of the transverse sinus region following CSF delivery of OVA-A594 (2.5 µL of 1 mg/mL) one month after CSF delivery of AA9-CMV-GFP or muT (3 μL of 1x10^13^ GC/mL).**(C)** Flow cytometry gating strategy for the identification of dural immune populations. **(D-F)** Flow cytometry and quantification of CSF-delivered OVA-A594 (2.5 µL of 1 mg/mL) uptake one hour post injection in dural macrophages and dendritic cells (DCs) one month after CSF delivery of AA9-CMV-GFP or muT (3 μL of 1x10^13^ GC/mL. ***, P<0.001 (Two-way ANOVA with Sidak’s post hoc test), *n* = 5 mice per AAV. **(G)** Quantification of immune cell frequency (% of all CD45^+^ cells) in the dura one month post CSF delivery of AA9-CMV-GFP or muT (3 μL of 1x10^13^ GC/mL). *, *P* < 0.05; ***, *P* < 0.001 (Two-way ANOVA with Sidak’s post hoc test), *n* = 5 mice per AAV. **(H-J) **Immunohistochemistry and quantification for Lyve1^+^ lymphatics and OVA-A594 drainage (2.5 µL of 1 mg/mL stock) one hour post injection one month after CSF delivery of AAV9-GFP or AAV9-muT (3 µL of 1x10^13^ GC/mL). **, *P* < 0.01; NS,  *P* >0.05 (Student’s t-test),*n* = 10 mice per AAV

Having developed a model allowing us to recapitulate ECM deposition around dural sinus regions harboring meningeal lymphatics, we sought to determine the consequences of this on CSF clearance routes critical to waste removal, namely glymphatic function, CSF efflux to dura mater, and meningeal lymphatic drainage. To achieve this, we performed CSF injections of AAV9-GFP or muT and one month later, glymphatic influx into the brain, CSF efflux to the dura mater, and CSF drainage to dCLNs was examined via CSF delivery of OVA-A594 (Fig. 4A). AAV9-muT-mediated transduction was efficient at driving Col1a1 induction in dural fibroblasts after seven days in vitro (Fig. 3J), however, the one-month time point in vivo was required to probe the effects of chronic collagen deposition on lymphatic vessels, the dural immune environment, and CSF clearance, more comparable to what would be observed in aging. Using immunostaining of CSF-access points in the dura we saw attenuated OVA-A594 accumulation, and these data were recapitulated using flow cytometry analysis of OVA-A594 uptake in dural antigen presenting cells, including macrophages and dendritic cells (Fig. 4B-F). Given that OVA-A594 accumulation is predominantly intracellular in these phagocytes, it is unclear whether these findings reflect attenuated uptake of CSF tracers by dural immune cells, or altered CSF access, but fibrosis and tissue stiffening has been previously observed to disrupt macrophage phagocytic capacity [33, 34]. We also observed enhanced dural macrophage numbers in this model, suggesting functional alterations which may be driven by altered ECM environment (Fig. 4G).

Multiple lymphatic networks participate in CSF drainage, including those in the dura mater and nasopharyngeal plexus [6–9]. To determine whether dural ECM deposition alters overall CSF drainage, we analyzed OVA-A594 uptake in the dCLNs, one month after AAV9-GFP or muT transduction. Immunostaining on dCLNs revealed a significant decrease in tracer drainage in the muT mice compared to GFP controls, suggesting compromised meningeal lymphatic function (Fig. 4H-J). Notably, Lyve1 lymphatic area in the dCLNs was unchanged between GFP and muT mice, indicating that muT expression specifically impaired tracer drainage without altering the lymphatic coverage. No changes were observed in glymphatic function in this model (Fig. S3 I and J). Collectively, these data suggest that dural Col1a1 deposition impairs CSF clearance to dCLNs, phenocopying observations seen during aging.

### Tissue stiffness impairs meningeal lymphatic function

**Fig. 5:**
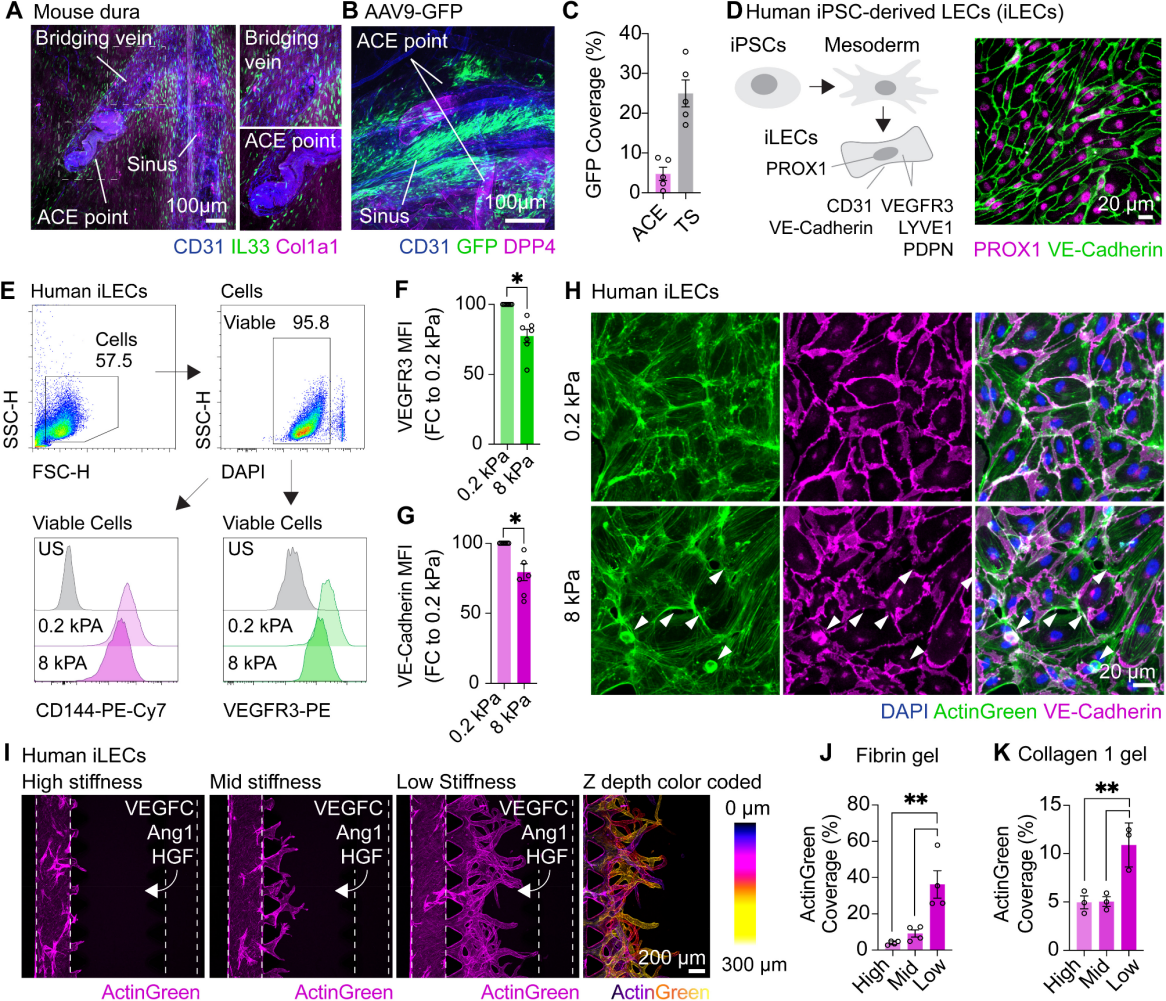
**Tissue stiffness impairs lymphatic function. (A) **Immunostaining of dural whole mounts showing abundant IL33^+^ fibroblasts surrounding bridging veins around dural sinuses but limited presence around arachnoid cuff exit (ACE) points. **(B, C)** Immunostaining and quantification for GFP coverage around dural sinuses or DPP4^+^ ACE points following CSF delivered AAV9-GFP expression (3 µL of 1x10^13^ GC/mL of GFP) one month post, *n* = 5 mice. **(D)** Summary schematic for the development of induced pluripotent stem cell (iPSC)-derived lymphatic endothelial cells (iLECS). iLECs express key lymphatic markers including PROX1 and VE-Cadherin. **(E-G) **Flow cytometry and quantification for surface staining of VE-cadherin and VEGFR3 72 hours after culturing on specially-formatted stiffness plates (0.2 kPA and 8 kPA). *,  *P *< 0.05 Wilcoxon matched-pairs signed rank test, *n* = 6 independent iLEC differentiations from *n* = 3 iPSC lines. **(H)** Immunocytochemistry of VE-cadherin in iLECS 72 hours after culturing on stiffness plates (0.2 kPA and 8 kPA). White arrows indicate regions of VE-Cadherin disruption in stiff plates. **(I) **iLECs labelled with ActinGreen sprouting on identX microfluidic chips in low stiffness (5 mg/mL fibrinogen to 4 U/mL thrombin), medium stiffness (25 mg/mL fibrinogen to 10 U/mL thrombin) and stiff stiffness (50 mg/mL fibrinogen to 20 U/mL thrombin) fibroin/thrombin gels towards lymphangiogenic factors (VEGF-C, angiopoietin 1 (Ang1) and hepatocyte growth factor (HGF), 100 ng/mL each) for 6 days. Z-depth colour coding shows vessels sprouting in 3-D. **(J, K) **Quantification of ActinGreen coverage of iLEC sprouting in variable stiffness fibrin/thrombin or Collagen 1 (low stiffness 1 mg/mL, medium stiffness 2 mg/mL or stiff stiffness 4 mg/mL) gels. **, *P* < 0.01 One way ANOVA with Dunnett’s post hoc test, *n* = 3/4 independent iLEC differentiations from *n* = 3 independent iPSC lines

Given that Col1a1 deposition around meningeal lymphatics impaired CSF clearance, we next sought to investigate factors that could contribute to these deficits. As there were no overt structural changes in the lymphatic system in the dura, yet notable drainage deficits were observed, we speculated that the collagen remodeling in the muT mice may have altered the mechanical properties of the surrounding tissue, particularly through increased tissue stiffness, which may interfere with normal CSF fluid dynamics and lymphatic drainage. First, we queried whether fibrosis around arachnoid cuff exit (ACE)-points could contribute to impaired CSF access. However, while fibroblasts are abundant on bridging veins close to the sinus, relatively few are present at ACE points and GFP transduction was sparse here compared to other sinus regions making it unlikely that altered collagen deposition in these regions explains the CSF clearance deficits observed (Fig. 5A and B).

We then investigated whether ECM-related phenotypes, particularly tissue stiffness, could directly impair lymphatic function. To further explore the relationship between tissue stiffness and lymphatic function, we used human iPSCs to generate iPSC-derived lymphatic endothelial cells (iLECs) via a mesoderm progenitor state, using a modified protocol [35] (Fig. 5D). Immunostaining and flow cytometry analysis demonstrated that iLECs express all canonical LEC markers, including PROX1, Lyve1, Podoplanin, and VEGFR3, and pan-endothelial markers VE-Cadherin and CD31 [3] (Fig. 5D and Fig. S4 A-J). RNA-seq analysis of iLECs confirms the presence of LEC transcripts, including *Prox1*, *Lyve1*, *Flt4*, *Gata2*, *Foxc2*, *Sox18*, *Nr2f2*, pan-endothelial transcripts *Pecam1* and *Cdh5*, and gene ontology pathways relating to lymphangiogenesis and vascular development (Fig. S5 A-E).

Having established this iLEC model, we next examined how tissue stiffness affects lymphatic function. Since Col1a1 induction is directly correlated with tissue stiffening [24], we cultured iLECs on CytoSoft defined-elasticity plates formulated as soft (0.2 kPa) or stiff (8 kPA) substrates for 72 h and analyzed features known to compromise meningeal lymphatic function—VEGFR3 and VE-Cadherin expression [36–38]. VEGFR3 is a key lymphatic growth factor, and impairment of the VEGF-C-VEGFR3 axis in the meningeal lymphatics results in lymphatic regression [37, 38]. Culturing on stiff matrixes revealed a reduction of surface VEGFR3 expression compared to soft matrices (Fig. 5E and F). Similarly, we recently demonstrated that VE-Cadherin disruptions in meningeal lymphatics impair CSF drainage [36], and age-related disruptions in VE-Cadherin are evident in the meningeal lymphatics [8]. Like VEGFR3, culturing on stiff matrices attenuated cell-surface VE-Cadherin expression, and immunostaining revealed discontinuities in cell-cell VE-Cadherin junctions that would result in lymphatic hyperpermeability and reflect age-related lymphatic alterations (Fig. 5F and G). Finally, to investigate how substrate stiffness affects lymphangiogenesis, we utilized a 3D microfluidic lymphatic sprouting assay that forms fully lumenized lymphatic structures [36, 39, 40] (Fig. S5 F-J). Using this assay with both fibrin/thrombin and Collagen 1 gels, we found increased substrate stiffness significantly impaired lymphatic sprouting towards the lymphangiogenic factors VEGF-C, Ang1, and HGF (Fig. 5I-K). These data demonstrate that elevated tissue stiffness impairs key lymphatic functions that control CSF drainage and suggest overall utility in anti-fibrotic strategies to normalize CSF drainage in conditions associated with dural fibrosis or ECM reorganization.

## Discussion

Fibrosis is a pathological condition implicated in numerous diseases and remains a leading cause of death globally [23]. While extensively studied in organs like the lungs, liver, and heart, fibrosis within the CNS—and specifically in the dura—has received limited attention. A hallmark of fibrosis is desmoplasia, a fibrotic response characterized by excessive ECM deposition, tissue stiffening, and dysregulated cellular function. This process is driven by activated fibroblasts, which deposit large amounts of ECM proteins, altering tissue architecture and mechanics [21]. Our findings demonstrate that the deposition of ECM components, particularly type I collagen, is a key contributor to impaired CSF clearance. As dural fibrosis progresses, the excessive accumulation of collagen and other ECM proteins reinforces a stiff, fibrotic environment that impairs lymphatic drainage.

These results establish dural ECM as a pathological feature with broad relevance to neurological conditions, including aging, TBIs, and neurodegenerative diseases such as Parkinson’s and Alzheimer’s disease. Under normal conditions, the dura’s collagen-rich ECM provides structural integrity and facilitates waste clearance from the CSF to the dCLNs through the meningeal lymphatic network. However, our findings reveal that dural ECM deposition disrupts these clearance mechanisms, which would ultimately lead to an accumulation of pathological waste. These disruptions are especially problematic in aging and TBI, where compromised lymphatic drainage further accelerates neurological decline [12, 13]. Importantly, many works have shown that enhancing lymphatic drainage is sufficient to augment waste clearance of pathological proteins such as amyloid beta and tau from the brain in Alzheimer’s disease mouse models [13, 41–43]. Appropriate meningeal lymphatic function has also been linked with improved synaptic function in the aged brain through modulating microglial responses [44], and the alleviation of neuroinflammatory deficits in the brain following TBIs [12]. However, conflicting evidence for a direct role in lymphatic modulation for brain waste removal does exist [45]. Continued research in this area and the development of improved meningeal-lymphatic specific targeting methods should help reconcile these differences.

Multiple divergent pathways have been implicated in driving fibrosis throughout the body. Importantly, fibroblasts and the cellular and molecular mechanisms driving fibrosis and ECM remodeling are heterogeneous across different tissues, making it necessary to study these pathways in the specific cells and tissues of interest [23, 46]. Previous works studying dural transcriptional responses following TBIs identified TGFβ1 as a key mediator driving fibroblast activation after injury [15, 29]. Here, our analysis using human dural fibroblasts—a cellular model that enhances the translational relevance to humans—also revealed TGFβ1, a well-known pro-fibrotic cytokine, as a key mediator of dural fibrosis. TGFβ1 has a myriad of pro-fibrotic functions including promoting chemotaxis and proliferation of fibroblasts, differentiation of fibroblasts into myofibroblasts, as well as suppressing ECM degradation and promoting ECM deposition [47–50]. Myofibroblasts, which play a crucial role in ECM production, contribute to the continuous generation of TGFβ1, thereby sustaining the fibrotic response [51]. TGFβ1 activation in dural fibroblasts, parallels its well-documented roles in peripheral fibrosis and systemic diseases. These effects are particularly relevant in the context of aging, where we observed significant transcriptional and structural changes in the dura, including signatures of response to TGFβ1 in aged dural fibroblasts and collagen reorganization around lymphatic networks—mirroring fibrotic patterns seen in peripheral tissues such as the skin [25]. These parallels further suggest that the mechanisms underlying systemic and CNS fibrosis may share overlapping therapeutic targets.

Using our novel AAV model for dural-specific induction of ECM, we were able to isolate the effects of this from the broader consequences of aging and TBI. This model revealed that ECM deposition alone is sufficient to impair meningeal lymphatic drainage, highlighting its direct impact on CSF clearance and establishing it as a critical factor that not only arises from injury or disease but actively drives pathological processes. While our study primarily focuses on Col1a1 changes, it is important to recognize that TGFβ1 signaling regulates multiple downstream pathways that may influence ECM-driven pathology [46]. Given the complexity of ECM remodeling we opted for TGFβR1 activation rather than direct overexpression of a single collagen (e.g., Col1a1). Our approach may reflect the broader ECM changes observed in aging and after injury, as TGFβ1 signaling can induce a diverse range of ECM components beyond Col1a1, including other collagen subtypes and fibronectin. We believe our approach provides a more physiologically relevant model for studying ECM accumulation and its impact on CSF clearance. Future studies will be necessary to dissect the specific contributions of individual ECM components and further delineate the potential broader effects of TGFβR1 activation.

Importantly, our findings suggest that therapeutic strategies targeting ECM deposition could have significant utility in alleviating neurological dysfunction. Anti-fibrotic treatments may improve cognitive outcomes in both aging populations and TBI patients by enhancing meningeal lymphatic function, as lymphatic augmentation has already demonstrated benefits in these conditions [12, 13, 44]. Furthermore, the dura’s location outside the blood-brain barrier (BBB) provides a unique advantage for therapeutic targeting [52]. This accessibility simplifies drug discovery efforts and enhances the feasibility of translating anti-fibrotic therapies into clinical practice [53].

Our study focused on a specific region of the dura looking at the dorsal and lateral aspects; however, other sites of CSF drainage, such as lymphatics in the basal regions and the recently described nasopharyngeal lymphatic plexus, likely contribute to the observed clearance deficits [9]. The observed ECM deposition in the muT mice may not be exclusively attributable to fibrotic changes in the dorsal/lateral lymphatics and any changes in lymphatic drainage deficits may represent a cumulative effect across all the targeted sites by the AAVs. Despite the complexity of the various CSF pathways, all routes ultimately converge to the dCLNs, which was assessed to explore functional CSF drainage. Nevertheless, future studies should assess the fibrotic impact across these regions to provide a more comprehensive understanding of CNS-wide clearance mechanisms, though it would be expected that fibroblasts in these regions would be similarly targeted by our CSF-delivered AAV, and therefore fibrosis in these regions likely contributes to our observed effects. In addition to the aforementioned lymphatic routes, other CSF efflux routes exist, for example surrounding perineural sheathes, which could contribute to compensatory CSF clearance upon disruptions of meningeal lymphatics [54]. Further, recent works demonstrated that other lymphatic routes—particularly the nasopharyngeal route—can partially compensate for lymphatic outflow during altered physiological states [55]. The exact consequences of these altered CSF dynamics remain unclear, but impaired meningeal lymphatic drainage led to elevated CSF outflow resistance and delayed CSF-to-blood efflux despite the recruitment of the nasopharyngeal pathway [55]. This suggests that CSF retention of tracers and impaired removal from this compartment occurs when CNS draining meningeal lymphatics are perturbed.

Importantly, our iPSC-derived model also does not differentiate between lymphatics networks that would be affected by fibrosis-related tissue stiffness. While known transcriptional differences exist in lymphatics between tissues [13], no protocols or isolation techniques exist to specifically culture meningeal and/or nasopharyngeal lymphatics—especially from humans. Another important consideration is the use of iPSC models compared to primary or immortalized primary LECs lines that are typically cultured from the skin or lymph nodes. We chose an iPSC approach as it provided the scalability, reproducibility, and purity to perform and interpret lymphangiogenesis assays (compared to primary cells) without the potential for unrestricted growth (from immortalized lines) that would limit our interpretations of VEGF-C signaling in lymphangiogenesis assays. These models have the additional benefit of being able to generate the required number of cells for particular experiments on demand, which can alleviate difficulties present in receiving primary cell lines from commercial suppliers, and enable an alternative model for researchers who cannot access human tissue sources.

Our findings underscore the need to further investigate the signaling pathways driving fibrosis in the CNS, particularly in the context of neurodegenerative and trauma-related CNS disorders. While TGFβ1 emerges as a central mediator, other pathways likely contribute to dural fibrosis and warrant exploration. For example, the roles of inflammatory cytokines, immune cell infiltration, and mechanical stress on ECM remodeling are areas of growing interest and may uncover novel therapeutic targets. Identifying additional upstream regulators of fibrosis and downstream consequences on meningeal lymphatic function will be crucial for designing targeted interventions that restore CSF clearance and improve neurological outcomes. Such efforts may also reveal broader connections between dural fibrosis and other fibrotic conditions throughout the body, further guiding therapeutic strategies that address systemic drivers of disease. Additionally, exploring potential additive or synergistic effects of targeting ECM remodeling with additional approaches that can enhance age-related lymphatic deficits, including targeting T cell-derived cytokines, or viral mediated overexpression of lymphangiogenic growth factor VEGF-C may further augment CSF clearance.

## Conclusions

Our study establishes peri-lymphatic dural collagen deposition as a key pathological feature that significantly impairs meningeal lymphatic function and CSF clearance. By demonstrating that collagen deposition is sufficient to disrupt these essential processes, we highlight its potential role in driving pathological changes associated with aging, TBIs and neurodegenerative diseases such as Parkinson’s and Alzheimer’s disease. These findings broaden our understanding of the mechanisms underlying CSF clearance impairment and underscore the importance of investigating fibrosis as a therapeutic target. Importantly, as we highlight collagen accumulation around lymphatic networks as a detrimental factor in CSF drainage this could suggest exploring anti-fibrotic therapies, including targeting immune-derived factors, as possible interventions. In acute settings such as TBIs, these interventions could prevent the onset of fibrosis, while in chronic conditions and aging, they may reverse fibrotic remodeling to restore normal meningeal lymphatic function and improve neurological outcomes. Given the dura’s accessibility outside the BBB, targeting fibrosis directly offers a unique advantage, bypassing many of the delivery challenges associated with CNS drug development.

## Methods

### Animals

Male or female wild-type mice (C57BL/6J background) were bred in-house, purchased from the Jackson Laboratory (JAX000664; WT) or provided by the National Institute of Health/National Institute of Aging. Mice were housed in a temperature and humidity-controlled environment with a 12-hour light/dark cycle. They were provided with food and water *ad libitum*. Mice were used for experiments at 2–3 months (young) or 20–24 months (old) as described below. All mice were wild-type, and thus no backcrossing was performed. All mice were habituated for at least 72 h in the animal facility before the start of experimentation. All experimental comparisons with young (2–3 month old) mice were made using littermate controls. Aged mice (20–24 month old) were received from the National Institute of Health/National Institute of Aging and compared to young mice received from the Jackson Laboratory. Mice were housed in a temperature- and humidity-controlled environment with a 12 h light/dark cycle (7:00 am–7:00 pm) and were provided with regular rodent chow and sterilized water *ad libitum*. Unless stated otherwise, mice were tested at 2–3 months of age (young mice) or 20–24 months of age (old mice). Sample sizes were chosen on the basis of a power analysis using estimates from previously published experiments [16, 36]. All experiments were approved by the Animal Ethics Committee at the University of Auckland, or the Institutional Animal Care and Use Committee of Washington University in St. Louis.

### Intra-cisterna magna injections

Mice were anesthetized via intraperitoneal (IP) ketamine (75 mg/kg) and medetomidine (1 mg/kg). The fur from the head and neck regions was removed with depilatory cream and the underlying skin was disinfected with 2% chlorhexidine with 70% ethanol. To prevent drying, an ophthalmic solution was applied to the eyes, and the head was securely fixed in a stereotaxic frame. Mice then received a subcutaneous injection of 2 mg/kg Bupivacaine for post-operative analgesia overlying the intended incision site. A small incision in the neck was made and the muscle layers were retracted with fine surgical hooks exposing the CSF-filled cisterna magna. Using a Hamilton syringe coupled to a 33-gauge needle, injections of 3 μL AAV9-CMV-mTGFRβ1, 3 μL of AAV9-CMV-GFP control (both at 10^13^ genome copies per mL in sterile PBS), or 2.5 μL of ovalbumin conjugated to Alexa Fluor 594 (OVA-A594; 1 mg/mL in sterile PBS) were administered into the CSF via an intra-cisterna magna injection at a controlled rate of 1 μL/min, with an additional 2-minute retention to prevent backflow. The syringe was carefully retracted, and the incision site was closed with Leukosan Adhesive Skin Glue. Following AAV injections, mice were subcutaneously administered 2.5 mg/kg Atipamezole hydrochloride for anaesthesia reversal and allowed to recover on a heat pad until fully awake.

### Immunohistochemistry

Mice were euthanized using a lethal dose of 150 mg/kg Pentobarbitone via IP injection and then transcardially perfused with ice cold heparin (10 U/mL) in PBS for exsanguination. For lymph node collections, mice were positioned supine and dCLNs were collected via gentle retraction of the submandibular glands and collection laterally either side of the trachea with fine surgical forceps. Subsequently, mice were decapitated just posterior to the occipital bone, and the overlying skin and muscle were removed from the skull. Using fine surgical scissors, the skull cap containing the skull bone and the attached dura mater was then carefully removed by clockwise incisions, beginning and ending at the occipital bone. The brain was then removed from the cavity. For ACE point analysis, mice were first perfused with ice cold heparin (10 U/mL) in PBS, then 4% PFA, and the skull caps, containing the dura, were collected as described but with gentle downwards pressure in the skull when collecting to ensure good bridging vein collection. Lymph nodes were then drop fixed in 4% PFA for 24 h at 4 °C and washed in PBS. The fixed nodes were placed in 30% sucrose for another 24 h at 4 °C before embedding in optimal cutting temperature (OCT) and rapid freezing over dry ice. Frozen lymph nodes were cut (30 μm thick sections) in a cryostat and collected onto Superfrost slides. Roughly 10–20 sections were cut for each node, spanning the entirety of the node. Skull caps containing meningeal whole mounts were drop fixed in 4% PFA for 2 h, and the dura mater was gently peeled from the inner surface of the skull using fine surgical forceps and then placed in PBS until further use. Brains were drop fixed in 4% PFA for 48 h at 4 °C then placed in 30% sucrose in PBS until the brain had completely sunk, usually within 24–48 h, at 4 °C. The brains were then embedded in OCT reagent, and rapidly frozen over dry ice and stored at -20 °C. Coronal cryosections (40–100 μm thick sections) were cut using a cryostat and the resulting free-floating sections were preserved in PBS until ready for use. Free-floating brain sections and meningeal whole mounts were subjected to blocking and permeabilization for one hour at room temperature in 24-well plates, with continuous agitation, using donkey immunobuffer (PBS with 1% Donkey serum and 0.2% Tritton X-100) at room temperature. Slides containing lymph nodes were marked with the outline of a hydrophobic pen and blocked/permeabilized for 30 min with donkey immunobuffer at room temperature. Brains and meninges were then incubated with primary antibodies overnight at 4 °C with gentle agitation in 24-well plates, and lymph nodes on slides in a humidified chamber. The list of antibodies used can be found in (Table S1). The following day, all tissues were washed 3 times with PBS-T. Sections were then incubated with secondary antibodies and DAPI (1 μg/mL) at room temperature for 2 hours with agitation for brains and meninges, or on slides for lymph nodes. Finally, sections were washed with PBS-T and brains and meninges mounted on Superfrost Plus slides (Fisher Scientific) and coverslipped using Anti-Fade Fluorescence Mounting Medium.

### Sirius red staining

Fixed meningeal whole mounts were prepared as described above. Sections were placed in a mould containing OCT and tissue was flattened using fine paintbrushes. OCT blocks were frozen rapidly over dry ice, and 20 μm sections through the transverse sinus were cut using a cryostat and mounted directly on Superfrost slides. Sirius red was dissolved in saturated aqueous picric acid to produce a solution consisting of 0.1% Direct red. Tissue sections were covered with Sirius red solution for 1 hour at room temperature and then washed with deionized water to remove excess dye. Samples were then dehydrated through a graded series of ethanol (75%, 80%, 95% and 100%) for 5 min each and lastly cleared in xylene solutions, 10 min each. Tissue was coverslipped with DPX mounting medium.

### Fast Green staining

Collagen staining using Fast Green dye was performed, with minor modifications, as described previously [56]. Whole mount meningeal preps were prepared as described above and mounted onto Superfrost glass slides and left for 2 hours at room temperature to dry onto the slide. Tissue was then dehydrated in a series of ethanol (30%, 50%, 70% and 100%) for 30 min each, and left for 1 hour in 100% methanol for an additional hour to ensure dehydration. Samples were then incubated on the slide with 2 μg/mL Fast Green for 2 hours at room temperature and washed with 100% methanol. Tissue was coverslipped with DPX mounting medium.

### Imaging and analysis

Widefield images were acquired using a Slideview VS200 slide scanner equipped with a 4x objective lens for macroscopic overview images and the 20 × (0.5 NA UPLFLN) objective lens was used for high resolution images. Confocal imaging was performed using the Leica LSM800 and 40x objective. Image analysis was performed using FIJI plugin for ImageJ (National Institutes of Health). Analysis of glymphatic influx was performed as described previously [36] using thresholded OVA-A594 coverage in brain sections as a percentage of overall brain-slice area. For each mouse, 8–12 brain sections (100 μm each) were analyzed and averaged to generate the value for a single mouse. Analysis of meningeal Lyve1, OVA, GFP or Col1a1 coverage was performed as described previously [36] using thresholded coverage as a percentage of the overall meningeal area. Peri-lymphatic Col1a1 was determined by generating a binary Lyve1 mask, expanding this by 10 μm, and then analyzing thresholded collagen coverage within this mask. Analysis of lymphatic drainage and Lyve1 coverage in lymph nodes was performed as described previously [36] using thresholded OVA or Lyve1 coverage as a percentage of overall lymph node area. For each mouse, 10–20 sections were analyzed from each lymph node and averaged to generate the value for a single mouse.

### Single cell isolation and flow cytometry

Unfixed dural meninges were removed from the skull cap using fine forceps and kept in ice-cold DMEM throughout the collection process. The meninges were digested for 15 min at 37 °C with constant agitation using 1 mL of pre-warmed digestion buffer (DMEM, 2% FBS, 1 mg/mL collagenase VIII, and 0.5 mg/mL DNase I). At completion, samples were triturated 5 times with a 1 mL pipette tip and filtered through a 70 μm cell strainer. Collagenase was then neutralized by the addition of 1 mL of DMEM containing 10% FBS. Following a centrifugation at 300 x g for 5 minutes, the samples were resuspended in ice-cold fluorescence-activated cell sorting (FACS) buffer (PBS with 1 mM EDTA and 1% BSA) and kept on ice until use. For surface staining, single cell suspensions were incubated in FACS buffer containing anti-CD16/32 (FC block), diluted 1:50 for 5 minutes, followed by the addition of fluorescently conjugated antibodies for 20 min at 4 °C. Subsequently, the samples were washed in FACS buffer and ran on a Cytek Aurora 5 laser system (School of Biological Sciences, UoA) and analyzed using FlowJo software (Tree Star). The list of antibodies used for flow cytometry can be found in (Table S1).

### Liquid chromatography mass spectrometry (LC-MS)

The methods for unlabelled LC-MS are detailed previously [16]. For TMT-labelled LC-MS, methods are described below.

### Peptide preparation

Peptides were prepared from solubilized mouse dura. Samples were digested as previously described [16]. Lysates were digested in 100 mM Tris-HCl buffer, pH 8, containing 4% sodium dodecyl sulfate, reduced with 100 mM dithiothreitol, and heated to 95 °C for 10 min. Nine μl of each sample was combined to make the reference pool sample. Reduced samples were mixed with 200 μl 100 mM Tris-HCL buffer, pH 8.5 containing 8 M urea (UA buffer) and transferred to the top of a 30,000 molecular weight cut-off filter and centrifuged at 10,000 x g for 10 min. An additional 300 μl of UA buffer was added and the filter was spun for another 10 min. The flow through was discarded and the proteins were alkylated using 100 μl of 50 mM iodoacetamide in UA buffer. Iodoacetamide in UA buffer was added to the top chamber of the filtration unit. The samples were gyrated at 550 rpm using a Thermomixer at room temperature for 30 min in the dark. The filter was spun at 10,000 x g for 10 min and the flow through discarded. Unreacted iodoacetamide was washed through the filter with two additions of 200 μl of UA buffer, and centrifugation at 10,000 x g for 10 min after each buffer addition. The UA buffer was exchanged with digestion buffer (50 mM ammonium bicarbonate buffer). Two sequential additions of digestion buffer with centrifugation after each addition to the top chamber was performed. The filters were transferred to a new collection tube and samples were digested with a combination of lysyl endopeptidase (1 mAU per filter) and sequencing grade modified trypsin in digestion buffer on top of the filter for 2 hours and overnight at 37 °C. The filters were spun at 14,000 x g for 15 min to collect the peptides in the flow through. The filter was washed with 100 mM ammonium bicarbonate buffer and the wash was collected with the peptides. In preparation for desalting, peptides were acidified to 1% (vol/vol) TFA final concentration. Peptides were desalted using two micro-tips on a Beckman robot (Biomek NX). The peptides were eluted with 60% (vol/vol) acetonitrile in 0.1% (vol/vol) TFA and dried in a Speed-Vac (Thermo Scientific, Model No. Savant DNA 120 concentrator). Samples were dissolved in 20 μl of 1% (vol/vol) MeCN in water. An aliquot (10%) was removed for quantification using the Pierce Quantitative Fluorometric Peptide Assay kit (Thermo Scientific). 1 μg total peptide from each sample and reference pool sample was aliquoted, lyophilized and stored at -80 ºC for TMT labelling.

### Preparation of TMT-labelled peptides

The lyophilized peptides (1 μg) from 5 bioreplicates of each condition and the reference pool sample were dissolved in 20 μl of HEPES buffer (100 mM, pH 8.5) and labelled according to the vendor protocol using the TMT-11 reagent kit (ThermoFisher Scientific). The labelled samples were combined, dried, and dissolved in 120 μl of 1% (vol/vol) formic acid (FA) in preparation for desalting. The TMT-11 labelled sample was desalted using PGC tips as described above.

### Ultra-high performance liquid chromatography mass spectrometry

The labeled peptides were analyzed using high-resolution nano-liquid chromatography tandem mass spectrometry (LC-MS). Chromatography was performed with an Acclaim PepMap 1000 C18 RSLC column (75 μm i.d. × 50 cm) (Thermo-Fisher Scientific) on an EASY-nanoLC 1000 (Thermo Fisher Scientific). The column was equilibrated with 11 μl of solvent A (1% (vol/vol) FA) at 700 bar pressure. The samples in 1% (vol/vol) FA were loaded (2.5 μl) onto the column with 1% (vol/vol) FA at 700 bar. Peptide chromatography was initiated with mobile phase A (1% FA) containing 5% solvent B (100% ACN, 1% FA) for 1 min, then increased to 25% B over 195 min, to 35% B over 40 min, to 70% B over 6 min, to 95% B over 2 min and held at 95% B for 18 min, with a flow rate of 250 nL/min. Data were acquired in data-dependent mode. Full-scan mass spectra were acquired with the Orbitrap mass analyser using a scan range of m/z = 375 to 1500 and a mass resolving power set to 70,000. Twelve data-dependent high-energy collisional dissociations were performed with a mass resolving power at 35,000, a fixed lower value of m/z 100, an isolation width of 1.2 Da, and a normalized collision energy setting of 32. The maximum injection time was 60 ms for parent-ion analysis and 120 ms for product-ion analysis. Ions that were selected for MS/MS were dynamically excluded for 40 s. The automatic gain control was set at a target value of 3 × 10^6^ ions for full MS scans and 1 × 10^5^ ions for MS2.

### Identification of and quantification of proteins

The machine data from the LC-MS analysis of isobarically-labelled peptides, using the Q-Exactive mass spectrometer, were converted to peak lists using Proteome Discoverer (version 2.1.0.81, ThermoScientific). MS2 spectra with parent ion charge states of ^+ 2, +3^ and ^+ 4^ were analyzed using Mascot software (Matrix Science, ). The MS data were searched using Mascot software against a concatenated UniProt database mouse (17,033 entries) and common contaminant proteins (cRAP, 116 entries). Trypsin/P enzyme specificity with a maximum of 4 missed cleavages allowed was used. The searches were performed with a fragment ion mass tolerance of 20 ppm and a parent ion tolerance of 20 ppm for Q Exactive™ data. PSMs were filtered at 1% false-discovery rate by searching against a reversed database. A minimum of two peptides with unique sequences, not resulting from missed cleavages, was required for identification of a protein. The processing, quality assurance and analysis of LC-MS data were performed with proteoQ, Mzion enables deep and precise identification of peptides in data-dependent acquisition proteomics. The precursor intensities were converted to logarithmic ratios (base 2), relative to the average precursor intensity across all samples. Within each sample, Dixon’s outlier removals were carried out recursively for peptides with greater than two identifying PSM’s. The median of the ratios of PSM that could be assigned to the same peptide was first taken to represent the ratios of the incumbent peptide. The median of the ratios of peptides was then taken to represent the ratios of the inferred protein. To align protein ratios across samples, likelihood functions were first estimated for the log-ratios of proteins using finite mixture modelling, assuming two-component Gaussian mixtures. The ratio distributions were then aligned so that the maximum likelihood of log-ratios was centred at zero for each sample. Scaling normalization was performed to standardize the log-ratios of proteins across all samples. To reduce the influence of outliers from either log-ratios or reporter-ion intensities, the values between the 5th and 95th percentile of log-ratios and 5th and 95th percentile of intensity were used in the calculations of standard deviations.

### Human dural fibroblast culture

Biopsy human dural specimens were acquired from Auckland City Hospital. Additional patient information is presented in (Table S2). Before dissection, tissue was washed with PBS and dissected into small explant cultures ∼ 1–5 mm in size, using a sterile blade. These explants were then transplanted into a 6-well tissue culture plate containing a low volume of Dulbecco’s modified Eagle medium (DMEM)/F12, supplemented with 10% FBS and 1% penicillin, streptomycin, glutamine (PSG), herein referred to as fibroblast media. The low volume of fibroblast media facilitates the attachment of the tissue explants. The tissue explants were incubated overnight at 37 °C with a 95% air/5% CO_2_ atmosphere. The following day, once the explants had attached to the surface, a small amount of fibroblast media was added to prevent dehydration. The growth rate of human dural fibroblasts is case dependent; however, it typically takes one week for cells to initiate growth and an additional two weeks to reach a confluent monolayer. After reaching 90% confluence, the explants were carefully removed and transferred onto a new 6-well plate. Subsequently, the cells were trypsinized (0.25% trypsin-1 mM ethylenediaminetetraacetic acid) (EDTA) at 37 °C for approximately 5 min to facilitate detachment and then transferred to a flask to promote cell expansion. Cell cultures were grown in fibroblast media and incubated at 37 °C, in an atmosphere containing 5% CO_2_. Prior to utilization, all solutions were pre-warmed to 37 °C. Every two days, half of the cell culture media was changed until cells reached 90% confluency. All work was done aseptically in a cell culture biosafety cabinet. To harvest cells for experimental procedures, the fibroblast media was removed and flasks containing cells were washed twice with PBS before trypsinization at 37 °C for 5 min. Subsequently, an equal volume of medium was introduced into the flask and gently mixed to dislodge any remaining adherent cells. The resulting suspension was then transferred to a Falcon tube and centrifuged at 300 x g for 5 min. The cell pellet was re-suspended in fibroblast media. Viable cell quantification was carried out by Trypan Blue exclusion with the aid of a haemocytometer. For a 96-well plate (Nunclon® delta, 0.33 cm^2^ per well) cells were seeded at a density of 10,000 cells per well. Cells were incubated overnight to allow attachment before experimental use. To establish a bank of dural fibroblast cells, cell expansion and cryopreservation was conducted by resuspending cell pellets in 90% FBS and 10% dimethyl sulfoxide (DMSO). The resulting cell suspension was aliquoted in vials, each containing 1 mL, at a final concentration of 1 million cells/mL. These vials were slowly cooled to -80 °C using a Mr. Frosty™ Freezing Container, before being transferred to liquid nitrogen for extended storage. When thawing, a cryovial was rapidly thawed in a 37 °C water bath. Subsequently, 10 mL of pre-warmed fibroblast media was gently added in a dropwise manner. The cell suspension was transferred to a T75 flask and incubated overnight at 37 °C to facilitate fibroblast attachment. The following day, a complete media change was carried out to remove non-adherent cells and to provide fresh media, enabling cell expansion and growth before using in future experiments.

### Mouse dural fibroblast culture

Adult female/male wild-type mice (2–3 months old) were euthanized humanely by CO_2_ inhalation. Mice were decapitated just posterior to the occipital bone, and the overlying skin and muscle were removed from the skull. Using fine surgical scissors, the skull cap was then carefully removed by clockwise incisions, beginning and ending at the occipital bone. The dural mater of the meninges were isolated by carefully peeling away from the skull cap using fine forceps and washed in fibroblast media. All procedures were performed in cell culture biosafety cabinet. The meninges then underwent a 15-minute digestion at 37 °C using 1 mL of pre-warmed digestion buffer (DMEM/F12 with 2% FBS, 1 mg/mL collagenase VIII from Sigma Aldrich, and 0.5 mg/mL DNase I from Sigma Aldrich). The digested material was triturated 5 times with a 1 mL pipette then filtered through a 70 μm cell strainer, and the collagenase was neutralized with 1 mL of fibroblast media. The resulting cell suspension was centrifuged at 300 x g for 5 min, and the cell pellet was resuspended and plated into a 6-well plate, with one meninge per well, in fibroblast media. The culture medium was changed every 3–4 days, and after 2-2.5 weeks, confluent cultures were observed. For passage or plating, cells were washed with PBS, dissociated using 0.25% Trypsin-EDTA for 5–10 min at 37 °C. The trypsin was neutralized with twice the volume of fibroblast media and centrifuged at 300 x g for 5 min. The cell pellet was then resuspended in fibroblast media and cells were counted using a haemocytometer and Trypan Blue exclusion. The fibroblasts were seeded at a density of 10,000 cells per well in 96-well plates.

### Fibroblast treatments

Dural fibroblasts were plated as aforementioned and allowed to reach 70–90% confluency before initiating treatments. Human dural fibroblasts were treated with cocktails containing 10 ng/mL of cytokines, as specified in (Table S3), for 48 h. The cytokines were reconstituted following the manufacturer’s instructions (Peprotech) and diluted in 0.1% bovine serum albumin (BSA). For mouse dural transduction, the AAV aliquots were thawed on ice, while confluent murine fibroblasts were trypsinized and plated following the aforementioned procedures. Once the cells were plated, the fibroblasts (still in suspension) were transduced with 1 × 10^10^ genome copies per mL (GC/mL) of the respective AAV (GFP or muT). Subsequently, the cells were incubated at 37 °C for one week.

### Human iPSC lines

The human iPSC lines (Table S4) were purchased from the American Type Culture Collection (ATTC) or the Jackson Laboratory. The use of iPSCs in this project was covered under HSNO approval number GMD102459.

### iPSC maintenance

iPSCs were maintained at 37 °C & 5% CO_2_ in StemFlex medium in Geltrex-coated plates (12.5 μg/cm^2^). The StemFlex medium was refreshed every two-three days. iPSC’s were passaged when ∼ 60–80% confluent. To passage, iPSCs were washed in PBS and brought to suspension with Versene. The iPSCs were gently triturated 3–5 times in the Versene solution until the cell suspension reached small clumps of ∼ 5–15 cells and iPSCs were split at a ratio of 1:6 − 1:40 depending on the line. StemFlex medium was refreshed the following day to remove Versene from the medium.

### LEC differentiation protocol

LECs were differentiated as described previously [35], but with minor modifications including adherent-based mesoderm induction [57] and the addition of IL-3 and Wnt5b in the LEC-induction media to drive PROX1 expression [58, 59]. When 60–80% confluent (day 0), iPSCs were brought into a single-cell suspension with Accutase. Accutase was inactivated with DMEM/F12 + 10% FBS (DMEM/F12 + 10% FBS), counted, and centrifuged at 200 x *g* for 4 min. iPSCs were resuspended in StemFlex medium + 10 μM ROCK inhibitor (Y-27632) and were seeded at 37,000 cells/cm^2^ into a Geltrex-coated 12- or 6-well plate and incubated at 37 °C & 5% CO_2_ for 24 h. On day 1, the StemFlex medium + Y-27,632 was replaced with Mesoderm Induction Media (MIM) (Table S5) for 3 days. On day 4, the cultures were placed in Lymphatic Induction Media (LIM) (Table S5). LIM was refreshed on day 5, generating a heterogenous cell culture on day 6, thus a magnetic-activated cell sorting (MACS)-based enrichment step was performed. On day 6, cells were brought into a single-cell suspension with Accutase, which was subsequently inactivated with DMEM/F12 + 10% FBS. The inactivated cell suspension was passed through a 70 μm cell strainer and washed through with an additional 2 mL of DMEM/F12. Cells were centrifuged at 200 x *g* for 4 min and the MACS enrichment was carried out using a MACS MultiStand, MidiMACS separator and a CD31 Microbead kit according to manufacturer’s instructions. Briefly, the supernatant was completely aspirated and the cell pellet was resuspended in 60 μL of DMEM/F12 followed by 20 μL of FcR Blocking reagent and 20 μL of CD31 Microbeads. The cell suspension was gently resuspended and was incubated at 4 °C for 15 min. Following the incubation, 1 mL of DMEM/F12 was added and the cell suspension was centrifuged at 200 x *g* for 4 min. During this process, an LS column was prepared by washing through 3 mL of DMEM/F12 in the magnetic field of a MidiMACS separator. The cell pellet was resuspended in 1 mL DMEM/F12 and was added to the prepared column. Once the solution had fully flowed through, the column was washed three times, each with 3 mL DMEM/F12. After the last wash, the LS column was removed from the separator, placed on top of a collection tube, and 5 mL of DMEM/F12 was added and immediately flushed through with a plunger. The CD31^+^ cell suspension was centrifuged at 200 x *g* for 4 min and resuspended in VEGF-C (100 ng/mL) supplemented endothelial media (sEM; ScienCell; Table S5). The CD31^+^ cells were plated in 0.1% gelatin coated plates at 150,000 cells/cm^2^.

### iLEC passaging and maintenance

iLECs were maintained at 37 °C & 5% CO_2_ in sEM which was refreshed every 3–4 days. iLECs were passaged when ∼ 60–90% confluent. To passage, cells were brought into single-cell suspension with trypsin. Trypsin was neutralized with DMEM/F12 + 10% FBS and cells were centrifuged at 300 x *g* for 5 min. Viable cells were resuspended in sEM and plated into gelatin-coated plates at a ratio of 1:2 − 1:3 for further expansion, or seeded at specific cell densities for each experiment. Where not otherwise mentioned, iLECs were seeded at 10,000 cells/well in 96 well plates and 15,000 cells/well in 48 well plates. All iLECs used in experiments were between passages 2–5, as cell senescence was observed thereafter.

### Cytosoft defined elasticity plates

iLECs were plated at 10,000 cells/well on Collagen 1-coated plates, as per manufactures instructions. For collagen coating, 100 μg/mL Collagen 1 was added to wells, incubated at room temperature for 1 h, and washed with PBS before cell addition. iLECs were incubated on plates for 72 h at 37 °C & 5% CO_2_.

### Microfluidic sprouting assays

identX microfluidic chips (Aim Biotech) and 10 μL pipette tips were pre-cooled at at 4 °C to minimize premature gelling. Fibrin/thrombin gels were prepared by the addition of soft (5 mg/mL fibrinogen to 4 U/mL thrombin), medium (25 mg/mL fibrinogen to 10 U/mL thrombin) and stiff (50 mg/mL fibrinogen to 20 U/mL) all on ice. Collagen gels were generated as soft (1 mg/mL), medium (2 mg/mL) or stiff (4 mg/mL) gels by addition of NaOH, H_2_O and 10X PBS as per (Table S6). The central gel chamber of identX microfluidic chips were loaded with 10 μL of respective gels using cooled chips and pipette tips. Chips were incubated at 37 °C for 30 min to enable gelling. The media chamber destined for iLEC seeding was coated with 15 μL of 0.1% gelatin-based coating solution for 5 min at RT, and the other media channel was hydrated with 15 μL sEM. The gelatin-based coating solution was washed and removed by addition of 70 μL to one media port, and 50 μL to the opposite attached media port. This was repeated three times, each time removing excess media. Media ports were then hydrated with 70 μL of sEM in one port, and 50 μL of sEM in the opposite attached port. iLECs were seeded in the cell gelatin-coated chamber at 60,000 cells/well in 20 μL sEM and allowed to adhere overnight. The next day, sEM was removed from all ports, and the iLEC-containing media ports received non supplemented EM (50 μL one port, 30 μL the other port) containing only the based formulation without FBS or ECGS, while the opposite media ports received complete EM further supplemented with 100 ng/mL VEGFC, Ang1, HGF (70 μL one port, 50 μL the other port). This approach generates a concentration gradient of lymphangiogenic factors to promote sprouting, and a small pressure difference to generate interstitial flow. This approach was repeated daily for 7 days. Chips were stained with Actin Green (2 drops/mL) and DAPI (1 μg/mL) for 2 h at room temperature, then washed with PBS-T, all via addition of 50 μL one port, 30 μL the other port on one side, and 70 μL one port, 50 μL the other port on the other side to visualize lymphatic structures.

### Immunocytochemistry

Cell cultures were fixed with 4% PFA at room temperature for 15 min. This was followed by three washes with PBS-T to permeabilize the cells for subsequent staining. Primary antibodies were diluted accordingly in PBS containing 0.2% Triton X-100, 1% donkey serum, and 0.05% proclin (collectively referred to as donkey immunobuffer). Cells were incubated overnight at 4 °C with gentle agitation. The next day, cells were washed three times with PBS-T for a total of 15 min before addition of secondary antibodies. Secondary antibodies and nuclear stain DAPI (1 μg/mL) were diluted in donkey immunobuffer and were added for two hours at room temperature with gentle agitation. Antibodies used can be found in (Table S1).

### Sirius red staining of cells

Cell cultures were fixed as described above. Cells were treated with 0.1% Sirius red in saturated picric acid solution and incubated overnight at 4 °C with gentle agitation. The following day, cells were washed three times with PBS-T for a total of 15 min to remove unbound dye. Then, cells were treated with 1 M NaOH at room temperature for 15 min with gentle agitation to dissolve the collagen dye complex.

### Image capture and analysis

The ImageXpress Micro® XLS high content screening system and Operetta® CLS high content screening system were used to capture fluorescent images of cultured cells in 96-well plates. The 20 × (0.45 NA) objective lens was used. The exposure times and focus were optimized for each plate to account for antibody efficiency. The quantitative analysis of image measurements was conducted with the FIJI package for ImageJ. For the coverage measurements of Col1a1, fibronectin and Sirius red data, positive signals were identified by thresholding of images, and quantified as area of signal/total area of site, resulting in a percentage area measurement. The same parameters were applied for across all groups during imaging and threshold settings, to mitigate experimental bias. The average percentage area for each condition was normalized relative to the average cell count, determined by the number of DAPI positive cells. Given the varied baseline expression of each parameter across individual cases, data was normalized based on the vehicle control within each respective case. Cell scoring analysis was carried out using the MetaXpress in-built cell scoring software (version 6.5.5). Briefly, images from the DAPI channel served as a reference and scoring was conducted in comparison to the corresponding FITC image. The GFP signal’s minimum and maximum width were adjusted to exclude any auto-fluorescent debris while ensuring cells were counted individually. Additionally, the intensity above background was optimized to minimize background noise. The same parameters were applied for across all groups within each case, to mitigate experimental bias.

### RNA-sequencing bioinformatic analysis

*Data Quality Control*: After obtaining the raw data (fastq files), the quality of the original reads including sequencing error rate distribution and GC content distribution, was evaluated using FastQC. The original sequencing sequences contain low quality reads and adapter sequences. To ensure the quality of data analysis, raw reads were filtered to get clean reads, and the subsequent analysis was based on clean reads. Data filtering was carried out using fastp mainly including the removal of adapter sequences in the reads, the removal of reads with high proportion of N (N denotes the unascertained base information), and the removal of low-quality reads. *Read Mapping to a Reference Genome*: The reference genome index was created by the build-index function in HISAT2 software package with default options. Then the filtered clean reads were mapped to reference genome by HISAT2, and the position and gene characteristics were acquired. After the alignment, the generated SAM files were sorted to BAM files using samtools. *Quantification and Differential Expression Analysis.* FeatureCounts software of subread package was used to quantify transcripts and the gene expression levels using mapped reads’ positional information on the gene. The differential expression levels of the gene, as well as the expression level of each single gene were analyzed. DESeq2 was used to analyze the DEGs (differentially expressed genes). During the analysis, samples were grouped so that comparisons between groups as a control-treatment pairwise could be done later. During the process, Log Fold Change ≥ 1.5 and FDR < 0.05 are set as screening criteria. *GO and KEGG Enrichment Analysis.* Gene Ontology enrichment analysis is a set of the internationally standardized classification system of gene function description that attempts to identify GO terms that are significantly associated with differentially expressed protein coding genes. GO enrichment analysis (Biological Processes) for the differentially expressed genes were performed using the R package ClusterProfiler. GO terms with corrected *P* values less than 0.05 were considered significantly enriched for differentially expressed genes.

### Statistical analysis

Two-tailed unpaired Student’s t-tests were used for pairwise comparisons between two groups. One-way ANOVA, accompanied by appropriate multiple-comparison tests (as specified in the figure legends), was utilized to assess differences among three distinct groups. For comparisons of multiple factors, two-way ANOVA with appropriate multiple comparisons tests (stated in figure captions) were used. Data was always presented as mean ± s.e.m. All statistical tests were conducted in using GraphPad Prism (GraphPad Software, v.9.0.2). For statistical analysis of LC-MS, metric multidimensional scaling and principal component analysis of protein log_2_-ratios was performed with the base R function stats: cmdscale and stats: prcomp, respectively. Heat-map visualisation of protein log_2_-ratios was performed with heatmap (pheatmap: Pretty Heatmaps. R package version 1.0.12). Linear modellings were performed using the contrast fit approach in limma to assess the statistical significance in protein abundance differences between groups. Adjustments of *p*-values for multiple comparison were performed with Benjamini-Hochberg correction. *P*-values were annotated as follows: NS, *P* > 0.05, *, *P* < 0.05, **, *P* < 0.01, ***, *P* < 0.001.

## Electronic supplementary material

Below is the link to the electronic supplementary material.

Supplementary Material 1

Supplementary Material 2

## Data Availability

All data needed to evaluate the conclusions in the paper are present in the paper and/or the Supplementary Materials. The data underlying Fig. S5 A-D are available in the Gene Expression Omnibus under accession number GSE270293. The data underlying Fig. 1 K; Figs. 2, C and D and 3 D and E; and Fig. S1 A–F are openly available in the Gene Expression Omnibus under the accession number GSE161290. These data were derived from sources in the public domain, specifically Rustenhoven et al. (2021). All codes used to analyze single and bulk RNA-seq are available from authors upon reasonable request.

## Abbreviations

AAV: Adeno-associated virus
CNS: Central nervous system
dCLN: Deep cervical lymph node
ECM: Extracellular matrix
GFP: Green fluorescent protein
ICC: Immunocytochemistry
IHC: Immunohistochemistry
iPSC: Induced pluripotent stem cell
LC-MS: Liquid chromatography-mass spectrometry
LECs: Lymphatic endothelial cells
OVA: Ovalbumin
qRT-PCR: Quantitative reverse transcription polymerase chain reaction
RNA-seq: RNA sequencing
sc-RNA-seq: Single-cell RNA sequencing
TBI: Traumatic brain injury
TGFβ1: Transforming growth factor beta 1
TGFβR1: Transforming growth factor beta receptor 1
