# Supplementary material for: Age-related meningeal extracellular matrix remodeling compromises CNS lymphatic function
Source: J Neuroinflammation. 2025 Apr 17;22:109. doi: 10.1186/s12974-025-03436-0 (PMC12007191; doi:10.1186/s12974-025-03436-0)
Supplement: Supplementary file 1 — Supplementary Material 1 [file 12974_2025_3436_MOESM1_ESM.docx]

Fig. S1.

**
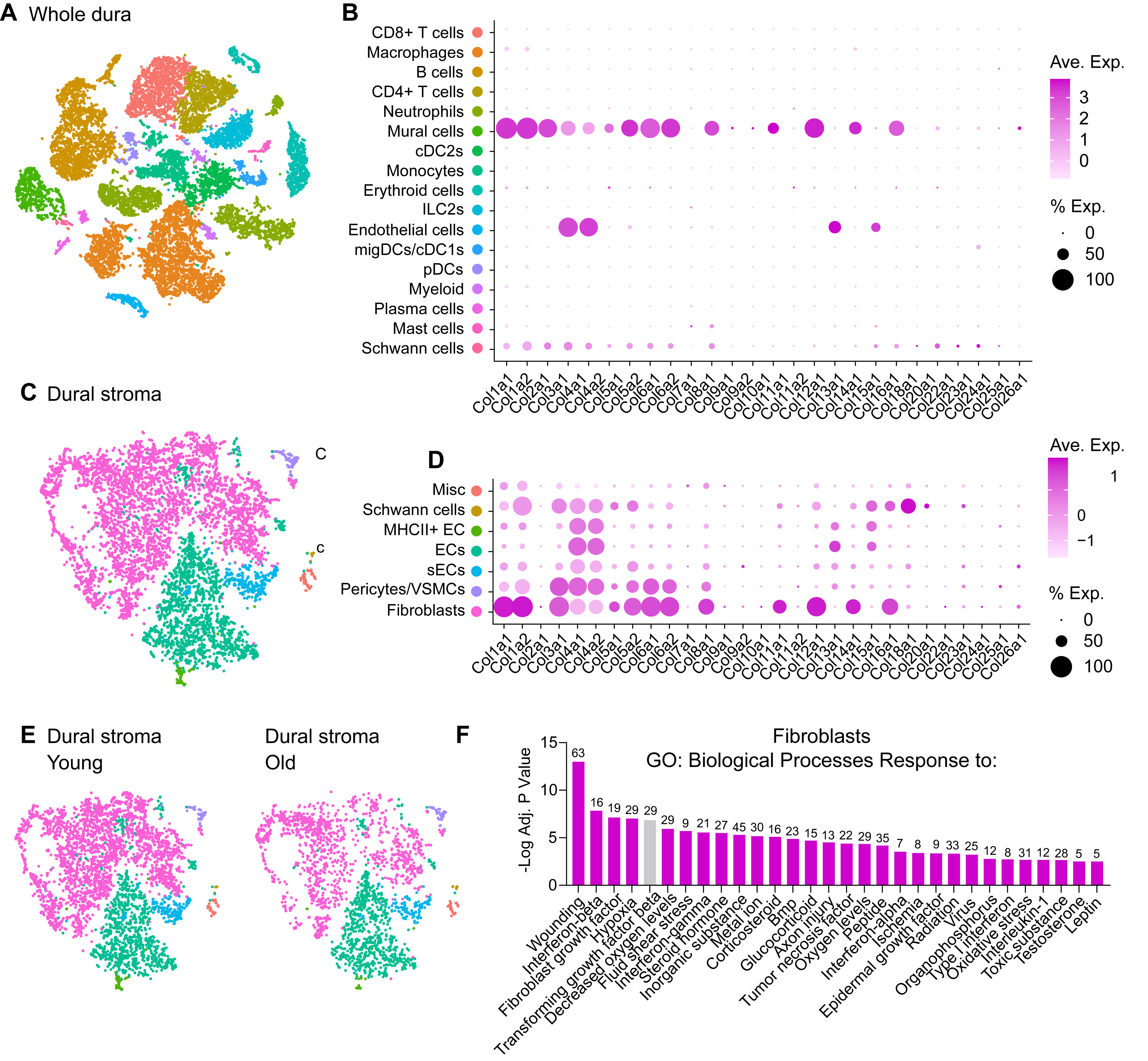
**

**Figure S1: Characterization of ECM production and fibrosis related signaling pathways in dural fibroblasts. (A)** t-SNE visualization of scRNA-seq of cells isolated from young (2–3 mo) and old (20–24 mo) mouse dura. Data from Rustenhoven et al. (2021); n = 5 individual young and old dura per experiment, n = 2 independent experiments, 10 dura samples per age total. **(B)** Dot plot showing scaled expression and percentage of cells expressing these genes in mouse dural stroma populations from reanalysis of scRNA-seq data in [16]. **(C)** t-SNE visualization of scRNA-seq of stromal (CD31^+^ and CD13^+^ sorted) cells isolated from young (2–3 mo) and old (20–24 mo) mouse dura. Data from Rustenhoven et al. (2021); n = 5 individual young and old dura per experiment, n = 2 independent experiments, 15 dura samples per age total. **(D)** Dot plot showing scaled expression and percentage of cells expressing these genes in mouse dural stroma populations from reanalysis of scRNA-seq data in [16]. **(E)** t-SNE visualization of scRNA-seq of stromal (CD31^+^ and CD13^+^ sorted) cells isolated from young (2–3 mo) and old (20–24 mo) mouse dura. Data from Rustenhoven et al. (2021); n = 5 individual young and old dura per experiment, n = 2 independent experiments, 15 dura samples per age total. **(F)** Analysis of upregulated gene ontology pathways for DEGs in old dural fibroblasts compared to young dural fibroblasts. Presented are the top “Biological Processes Response to” pathways.

Fig. S2.

**
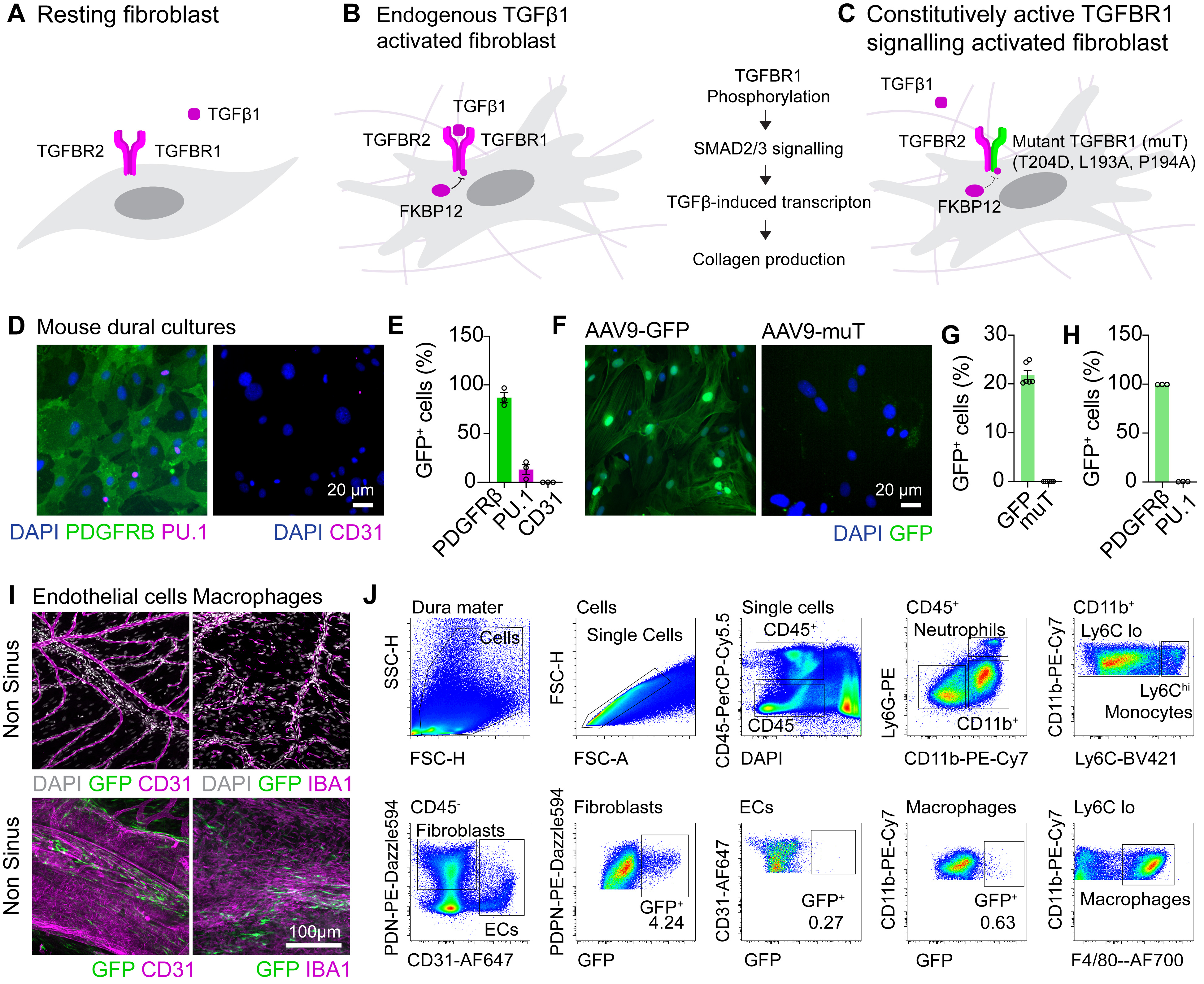
**

**Figure S2: Description of the muT fibrosis model and validation of AAV9 targeting in dural fibroblasts. (A-C)** Summary schematic for the use of the muT model to deliver a constitutively active TGFβR1 to fibroblasts to drive constitutive TGFβ1 signaling and collagen production. **(D, E)** Characterization of mouse dural cultures by immunostaining displaying predominantly PDGFRβ^+^ fibroblasts, some PU.1^+^ macrophages, and no CD31+ vasculature. **(F)** Immunocytochemistry and quantification for GFP expression in PDGFRβ^+^ fibroblasts or PU.1^+^ macrophages one week following AAV9 transduction with 1x10^10^ GC/mL AAV9-GFP or AAV9-muT, n = 6 independent mouse dura cultures per group. **(J)** Flow cytometry gating strategy for the identification of GFP expressing cells in dura one month after delivery of AA9-CMV-GFP (3 μL of 1x10^13^ GC/mL).

Fig. S3.

**
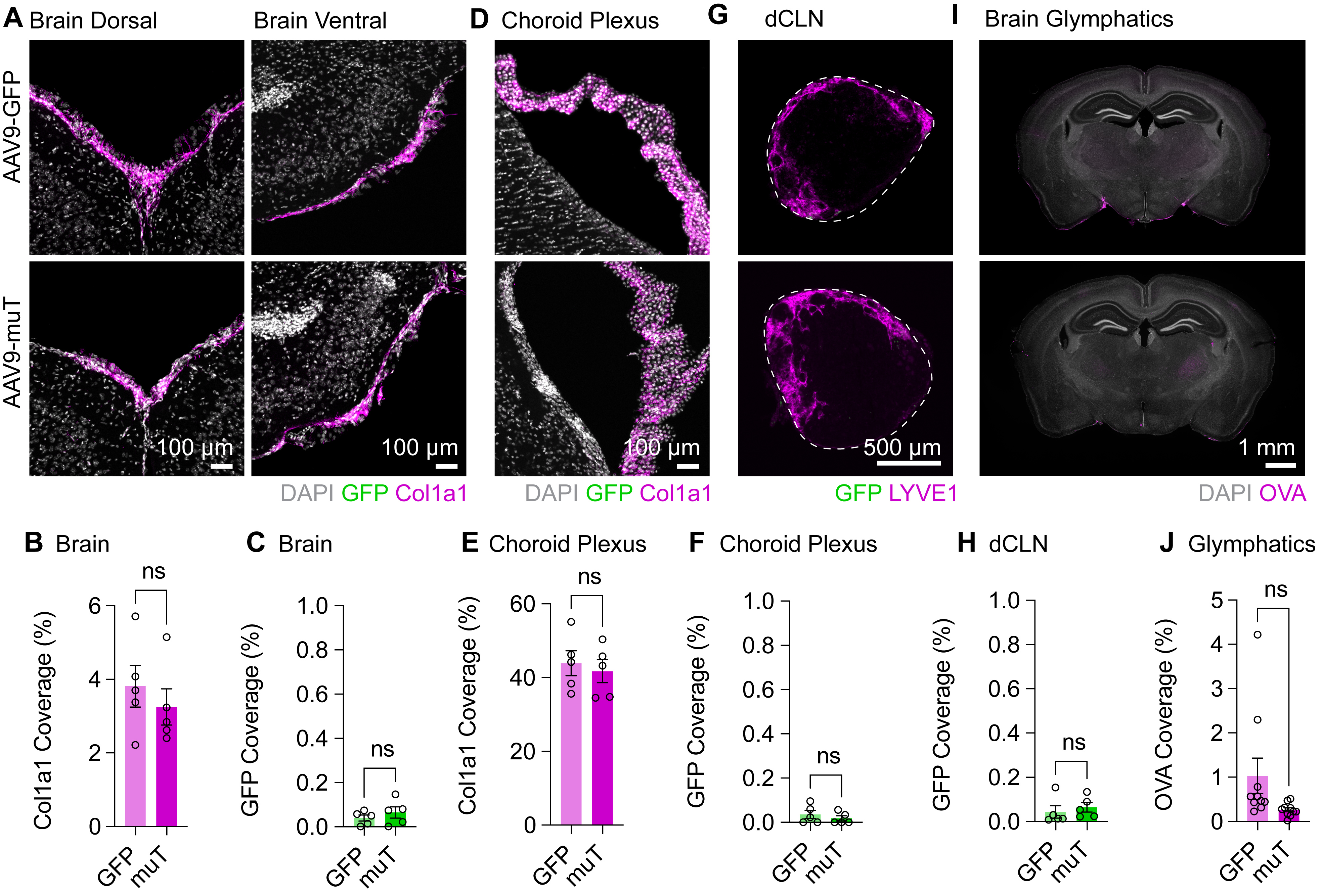
**

**Figure S3: Lack of AAV9-targetting in brain, leptomeningeal, or choroid plexus and glymphatic influx in the muT model. (A-C)** Immunohistochemistry and quantification for Col1a1 coverage and GFP expression in the brains/leptomeninges one month after CSF delivery of AAV9-GFP or AAV9-muT (3 µL of 1x10^13^ GC/mL). NS, P>0.05 (Student’s t-test), n = 5 mice per AAV. **(D-F)** Immunohistochemistry and quantification for Col1a1 coverage and GFP expression in the choroid plexus one month after CSF delivery of AAV9-GFP or AAV9-muT (3 µL of 1x10^13^ GC/mL). NS, P>0.05 (Student’s t-test), n = 5 mice per AAV. **(G, H)** Immunohistochemistry and quantification for GFP expression in the dCLNs one month after CSF delivery of AAV9-GFP or AAV9-muT (3 µL of 1x10^13^ GC/mL). NS, P>0.05 (Student’s t-test), n = 5 mice per AAV. **(J, H)** Immunohistochemistry and quantification for OVA-A594 drainage (2.5 µL of 1 mg/mL stock) one hour post injection one month after CSF delivery of AAV9-GFP or AAV9-muT (3 µL of 1x10^13^ GC/mL). NS, P>0.05 (Student’s t-test), n = 10 mice per AAV. **Fig. S4.**

**
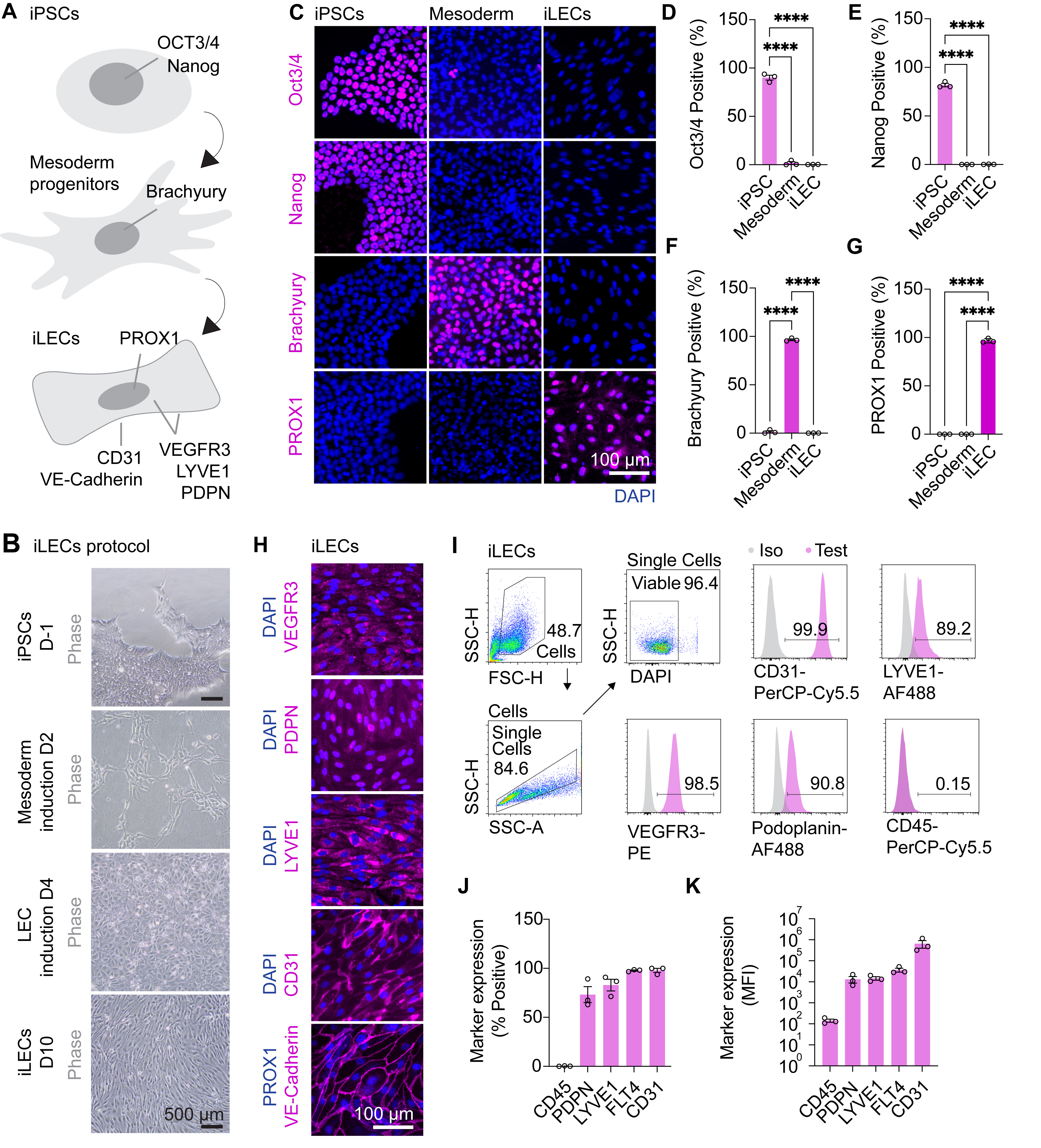
**

**Figure S4: Characterization of iPSC-derived LECs (iLECs).** **(A)** Summary schematic detailing the differentiation approach for the generation of iLECS from iPSCs via a mesoderm lineage and expected markers and localization at individual stages. **(B)** Phase contrast microscopy and immunocytochemistry detailing expected morphologies and phenotypes of cells at selected stages of differentiation. **(C-G)** Immunocytochemistry and quantification for state-specific markers in iPSCs, mesoderm progenitors, and iLECs. ****p<0.0001 one-way ANOVA with Tukey’s post-hoc test, n = 3 independent differentiations, from 3 different iPSC lines. **(H)** Immunocytochemistry for select LEC marker genes on iLECs. **(I-K)** Flow cytometry gating strategy and analysis and quantification of LEC markers on iLECs, n = 3 independent differentiations, from 3 different iPSC lines.

Fig. S5.


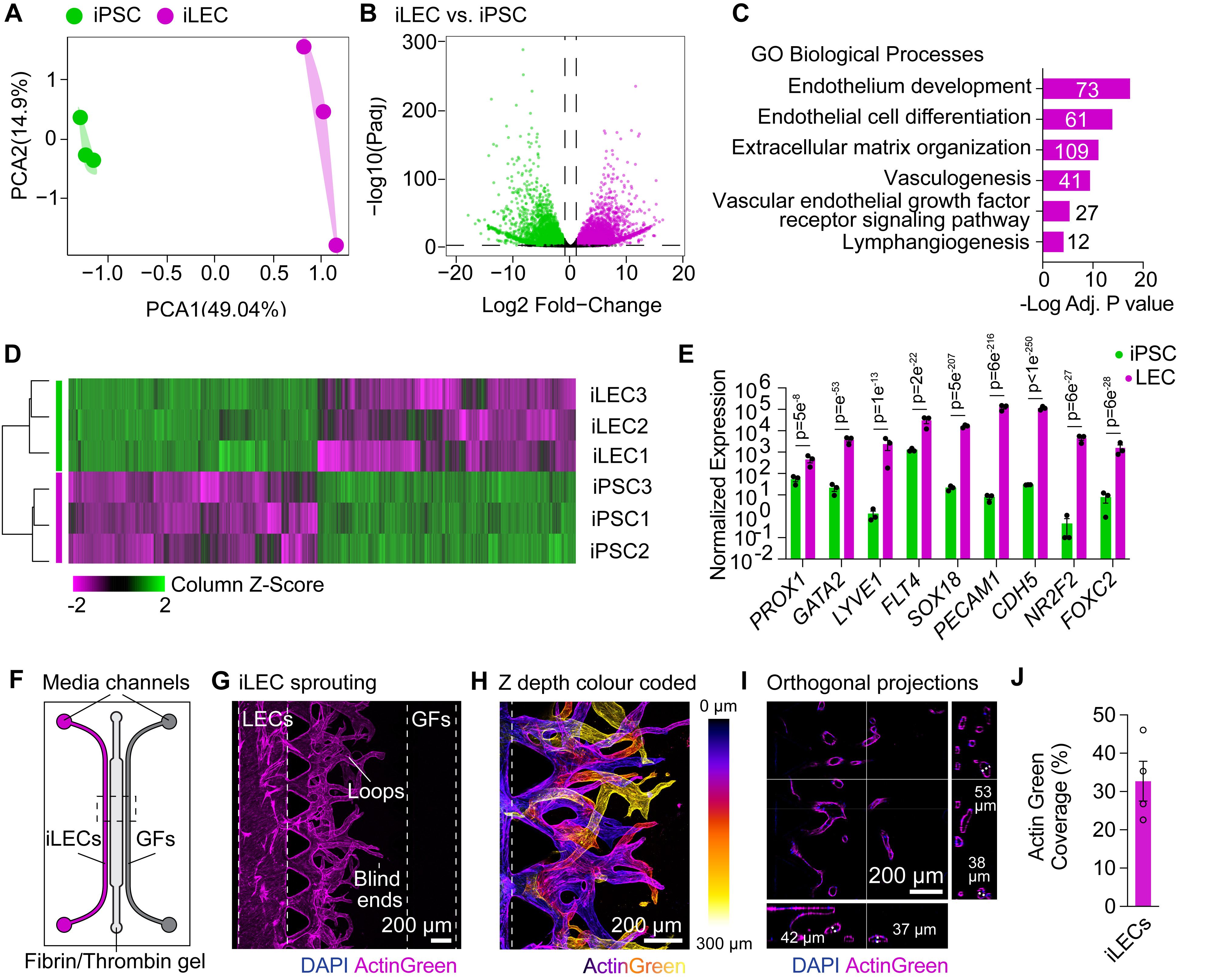


**Figure S5: RNA-seq validation of iLECs and characterization of the lymphangiogenesis sprouting assay. (A)** PCA plot showing clustering of iPSCs or iLECS based on the first two principal components from RNA-seq analysis. Data represent 3 independent differentiations, from 3 different iPSC lines. **(B)** Volcano plot showing significantly DEGs for iLECs compared to iPSCs. **(C)** Select GO biological processes enriched in iLECs compared to iPSCs. Numbers represent the number of genes involved. **(D)** Heatmap for DEGs showing unsupervised dendrogram clustering of iPSCs and iLECs. **(E)** Normalized expression values for canonical LEC marker genes, fate-defining transcription factors, and endothelial genes. P = False-discovery rate-adjusted p values. **(F)** Summary schematic for iLEC sprouting assays into a fibrin/thrombin gel using identX microfluidic devices. iLECs are seeded in the left media chamber, the fibrin/thrombin gel in the central chamber, and growth factors VEGF-C, Ang1, and HGF are applied to the right media chamber. GFs = growth factors. **(G)** ActinGreen staining for iLECs sprouting into fibrin/thrombin gels, 6 days after daily administration of 100 ng/mL VEGF-C, Ang1, and HGF to the right media chamber. GFs = growth factors. **(H)** Color coding of ActinGreen staining of iLEC sprouts into fibrin/thrombin gels, 6 days after daily administration of 100 ng/mL VEGF-C, Ang1, and HGF to the right media chamber, by confocal imaging based on Z-depth within the fibrin/thrombin gel. **(I)** Orthogonal projections of iLEC sprouts from (**H**) stained with ActinGreen and DAPI showing fully lumenized vessels with physiological diameters. Selected diameters of lymphatic vessels are shown. **(J)** Quantification of iLEC sprouting area by ActinGreen staining, n = 4 independent differentiations, from 2 different iPSC lines.
