# Supplementary material for: Age-related meningeal extracellular matrix remodeling compromises CNS lymphatic function
Source: J Neuroinflammation. 2025 Apr 17;22:109. doi: 10.1186/s12974-025-03436-0 (PMC12007191; doi:10.1186/s12974-025-03436-0)
Supplement: Supplementary file 2 — Supplementary Material 2 [file 12974_2025_3436_MOESM2_ESM.docx]

**Table S1.**

**List of antibodies used in this project.**

| Antigen | Species/Conjugate | Company | Cat. No. | ICC | IHC | FC |
| --- | --- | --- | --- | --- | --- | --- |
| α-SMA | Mouse | DAKO | Is611 | 1:10 |  |  |
| Brachyury | Rabbit | Abcam | Ab209665 | 1:500 |  |  |
| CD3e-eF660 | Rat | ThermoFisher | 50-0032-82 |  |  | 1:200 |
| CD4-FITC | Rat | ThermoFisher | 11-0041-82 |  |  | 1:200 |
| CD8a-APC-eF780 | Rat | ThermoFisher | 47-008-82 |  |  | 1:200 |
| CD11b-PE-Cy7 | Rat | ThermoFisher | 25-0112-82 |  |  | 1:200 |
| CD11c-BV605 | Armenian Hamster | BD Biosciences | 563057 |  |  | 1:200 |
| CD13 | Mouse | Biolegend | 301702 | 1:100 |  |  |
| CD16/32 | Rat | Biolegend | 101301 |  |  | 1:200 |
| CD19-BV480 | Rat | BD Biosciences | 566107 |  |  | 1:200 |
| CD31 | Goat | R & D Systems | AF3628 |  | 1:20 |  |
| CD31 | Mouse | DAKO | MO823 | 1:500 |  |  |
| CD31--PerCP-Cy5.5 | Mouse | Biolegend | 303132 |  |  | 1:200 |
| CD45 | Mouse | Abcam | Ab8216 | 1:500 |  |  |
| CD45 | Rat | ThermoFisher | 45-0451-82 |  |  | 1:200 |
| CD45-PerCP-Cy5.5 | Mouse | BD Bioscience | 564105 |  |  | 1:62 |
| Col1a1 | Mouse | Santa Cruz | SC293182 | 1:100 |  |  |
| Col1a1 | Rabbit | Abcam | Ab21286 |  | 1:250 |  |
| Anti-goat AF647 | Donkey | ThermoFisher | A21447 | 1:500 |  |  |
| Anti-mouse AF488 | Donkey | ThermoFisher | A21202 | 1:500 |  |  |
| Anti-mouse AF647 | Donkey | ThermoFisher | A31571 | 1:500 | 1:500 |  |
| Anti-rabbit AF488 | Donkey | ThermoFisher | A21206 | 1:500 |  |  |
| Anti-rabbit AF594 | Donkey | ThermoFisher | A21207 |  | 1:500 |  |
| Anti-rat AF594 | Donkey | ThermoFisher | A21209 |  | 1:500 |  |
| DPP4 | Mouse | R & D Systems | AF954 |  | 1:200 |  |
| F4/80-AF700 | Rat | ThermoFisher | 56-4801-82 |  |  | 1:200 |
| Fibronectin | Rabbit | DAKO | A0245 | 1:10,000 |  |  |
| HLA-DR | Mouse | DAKO | MO775 | 1:500 |  |  |
| IL-33 | Mouse | R & D Systems | AF3626 |  | 1:200 |  |
| IBA1 | Goat | Abcam | ab5076 |  | 1:200 |  |
| Ly6C-BV421 | Rat | BioLegend | 128031 |  |  | 1:200 |
| Ly6g-PE | Rat | ThermoFisher | 12-9668-82 |  |  | 1:200 |
| LYVE1-eF660 | Rat- | ThermoFisher | 50-0443-82 |  | 1:200 |  |
| LYVE1-AF488 | Rabbit | Novus Biologicals | 50-0443-82 | 1:20 |  | 1:125 |
| MHCII-PE-Cy5 | Rat | ThermoFisher | 15-5321-81 |  |  | 1:2500 |
| NK1.1-BV510 | Mouse | BioLegend | 108737 |  |  | 1:200 |
| Nanog | Mouse | BD Biosciences | 611202 | 1:500 |  |  |
| OCT3/4 | Mouse | BD Biosciences | 560482 | 1:500 |  |  |
| PDGFRα | Rabbit | Abcam | Ab203491 | 1:1,000 |  |  |
| PDGFRβ | Goat | R&D Systems | AF385 | 1:1,000 |  |  |
| Podoplanin-AF488 | Rat | ThermoFisher | 17-9381-42 | 1:20 |  | 1:32 |
| PROX1 | Rabbit | Abcam | Ab199359 | 1:100 |  |  |
| PU.1 | Rabbit | Cell Signalling | 2258 | 1:500 |  |  |
| TMEM119 | Rabbit | Abcam | Ab185333 | 1:500 |  |  |
| VE-Cadherin | Goat | R & D Systems | AF938 | 1:500 |  |  |
| VE-Cadherin-PE-Cy7 | Rat | Biolegend | 138016 |  |  | 1:200 |
| VEGFR3 | Mouse | R & D Systems | MAB3491 | 1:100 |  |  |
| VEGFR3-PE | PE | Biolegend | 356204 |  |  | 1:200 |
| Vimentin | Mouse | Santa Cruz | SC6260 | 1:500 |  |  |

**Table S2.**

**Patient demographics for dural fibroblasts**

| Case Number | Age | Sex | Confirmed Pathology |
| --- | --- | --- | --- |
| HGB0003 | 61 | M | Recurrent glioblastoma |
| HGB0004 | 68 | M | Subarachnoid haemorrhage to ruptured aneurysm |
| HGB0005 | 30 | M | Anaplastic astrocytoma |
| HGB0006 | 70 | M | Recurrent glioblastoma |
| HGB0007 | 44 | F | Recurrent glioblastoma |
| HGB0008 | 53 | F | Meningioma – Grade I |
| HGB0009 | 66 | M | Parkinson’s disease – deep brain stimulation |
| HGB0021 | 58 | F | Cortical dysplasia – ILAE type IIa |
| HGB0028 | 30 | F | Meningioma – Grade I |
| HGB0033 | 40 | F | Meningioma – Grade II |
| HGB0034 | 66 | F | Metastatic Adenocarcinoma – lung primary |

**Table S3.**

**List of cytokines used for fibrotic response.**

| Th cocktail | Cytokine (human) | Company | Catalogue number |
| --- | --- | --- | --- |
| Th1 | IFNγ | Peprotech | 300-02 |
| Th1 | TNFα | Peprotech | 300-01A |
| Th1 | IL-2 | Peprotech | 200-02 |
| Th2 | IL-4 | Peprotech | 200-04 |
| Th2 | IL-5 | Peprotech | 200-05 |
| Th2 | IL-6 | Peprotech | 200-06 |
| Th2 | IL-10 | Peprotech | 200-10 |
| Th2 | IL-13 | Peprotech | 200-13 |
| Th2 | IL-25 | Peprotech | 200-24 |
| Th17 | IL-17A | Peprotech | 200-17 |
| Th17 | IL-17F | Peprotech | 200-25 |
| Th17 | IL-21 | Peprotech | 200-21 |
| Th17 | IL-22 | Peprotech | 200-22 |
| Treg | TGFβ | Peprotech | 100-21 |

**Table S4.**

**iPSC lines used for LEC differentiation**

| iPSC Line | Source | Sex | Age | Ethnicity | Alias |
| --- | --- | --- | --- | --- | --- |
| ATCC-BYS0112 | ATCC | M | 31 | Non-Hispanic Caucasian | Line 1 |
| ATCC-BXS0116 | ATCC | F | 31 | Non-Hispanic Caucasian | Line 2 |
| KOLF2.1J | Jackson Laboratory | M | 55-59 | Non-Hispanic Caucasian | Line 3 |

**Table S5.**

**Media compositions used for LEC differentiation**

| Reagent | Stock Concentration | Final concentration/volume |
| --- | --- | --- |
| iPSC growth media | | |
| StemFlex | 1X | 1X |
| iPSC growth media—prior to mesoderm induction | | |
| StemFlex | 1X | 1X |
| Y-27632 (Dihydrochloride) | 10 mM | 10 µM |
| Mesoderm induction media | | |
| DMEM/F12 | 1X | 0.5X |
| Neurobasal | 1X | 0.5X |
| Glutamax | 100X | 1X |
| N2 | 50X | 1X |
| B27 | 50X | 1X |
| CHIR99021 | 3 mM | 8 µM |
| BMP4 | 100 µg/mL | 25 ng/mL |
| Lymphatic endothelial cell induction media | | |
| StemPro34 | 1X | 1X |
| Glutamax | 100X | 1X |
| Forskolin | 2 mM | 2 µM |
| VEGF-C | 100 µg/mL | 100 ng/mL |
| WNT5b | 100 µg/mL | 100 ng/mL |
| VEGF-A | 100 µg/mL | 20 ng/mL |
| Ang1 | 100 µg/mL | 20 ng/mL |
| BMP4 | 100 µg/mL | 20 ng/mL |
| IL3 | 100 µg/mL | 20 ng/mL |
| Lymphatic endothelial cell growth media | | |
| Endothelial Cell Medium | 1X | 1X |
| VEGF-C | 100 µg/mL | 100 ng/mL |

**Table S6.**

**Collagen gel formation**

| Component | Volume (Per gel) | Final amount |
| --- | --- | --- |
| Collagen 1 (~8 mg/mL) | 100 µL | 0.5 Parts |
| 0.1 M NaOH | 13.4 µL | 0.067 Parts |
| H_2_0 | 66.6 µL | 0.33 Parts |
| 10 X PBS | 20 µL | 0.1 Parts |
